# Microvascular aberrations found in human polycystic kidneys are an early feature in a *Pkd1* mutant mouse model

**DOI:** 10.1242/dmm.052024

**Published:** 2025-04-28

**Authors:** Daniyal J. Jafree, Charith Perera, Mary Ball, Daniele Tolomeo, Gideon Pomeranz, Laura Wilson, Benjamin Davis, William J. Mason, Eva Maria Funk, Maria Kolatsi-Joannou, Radu Polschi, Saif Malik, Benjamin J. Stewart, Karen L. Price, Hannah Mitchell, Reza Motallebzadeh, Yoshiharu Muto, Robert Lees, Sarah Needham, Dale Moulding, Jennie C. Chandler, Sonal Nandanwar, Claire L. Walsh, Paul J. D. Winyard, Peter J. Scambler, René Hägerling, Menna R. Clatworthy, Benjamin D. Humphreys, Mark F. Lythgoe, Simon Walker-Samuel, Adrian S. Woolf, David A. Long

**Affiliations:** ^1^ Developmental Biology and Cancer Research and Teaching Department, UCL Great Ormond Street Institute of Child Health, University College London, London WC1N 1EH, UK; ^2^ UCL Centre for Kidney and Bladder Health, University College London, London WC1E 6BT, UK; ^3^ Specialised Foundation Programme in Research, NHS East of England, Cambridge CB21 5XB, UK; ^4^ UCL Centre for Advanced Biomedical Imaging, University College London, London WC1E 6DD, UK; ^5^ Central Laser Facility, Science and Technologies Facilities Council, UK Research and Innovation, Didcot OX11 0QX, UK; ^6^ Lymphovascular Medicine and Translational 3D-Histopathology Research Group, Charité Universitätsmedizin Berlin, Berlin 10117, Germany; ^7^ Berlin Institute of Health at Charité-Universitätsmedizin Berlin, BIH Center for Regenerative Therapies, Berlin 10117, Germany; ^8^ Molecular Immunity Unit, Department of Medicine, University of Cambridge, Cambridge CB2 1TN, UK; ^9^ Wellcome Sanger Institute, Wellcome Genome Campus, Hinxton CB10 1SA, UK; ^10^ Mathematical Sciences Research Centre, Queen's University Belfast, Belfast BT7 1NN, UK; ^11^ Research Department of Surgical Biotechnology, Division of Surgery and Interventional Science, University College London, London NW3 2PF, UK; ^12^ UCL Institute of Immunity and Transplantation, University College London, London NW3 2PF, UK; ^13^ Division of Nephrology, Department of Medicine, Washington University in St Louis, St Louis, MO 63110, USA; ^14^ Department of Mechanical Engineering, University College London, London WC1E 7JE, UK; ^15^ School of Biological Sciences, Faculty of Biology Medicine and Health, University of Manchester, Manchester M13 9PT, UK

**Keywords:** Magnetic resonance imaging, Nephrology, Perfusion, Single-cell RNA sequencing, Three-dimensional microscopy, Vasculature

## Abstract

Therapies targeting blood vessels hold promise for autosomal dominant polycystic kidney disease (ADPKD), the most common inherited disorder causing kidney failure. However, the onset and nature of kidney vascular abnormalities in ADPKD are poorly defined. Accordingly, we employed a combination of single-cell transcriptomics; three-dimensional imaging with geometric, topological and fractal analyses; and multimodal magnetic resonance imaging with arterial spin labelling to investigate aberrant microvasculature in ADPKD kidneys. Within human ADPKD kidneys with advanced cystic pathology and excretory failure, we identified a molecularly distinct blood microvascular subpopulation, characterised by impaired angiogenic signalling and metabolic dysfunction, differing from endothelial injury profiles observed in non-cystic human kidney diseases. Next, *Pkd1* mutant mouse kidneys were examined postnatally, when cystic pathology is well established, but before excretory failure. An aberrant endothelial subpopulation was also detected, concurrent with reduced cortical blood perfusion. Disorganised kidney cortical microvasculature was also present in *Pkd1* mutant mouse fetal kidneys when tubular dilation begins. Thus, aberrant features of cystic kidney vasculature are harmonised between human and mouse ADPKD, supporting early targeting of the vasculature as a strategy to ameliorate ADPKD progression.

## Teaser

**Editor's choice:** A distinct subpopulation of aberrant microvasculature was identified in human polycystic kidneys. Similar abnormalities in mice, together with structural and functional microvascular changes, were found to precede excretory kidney failure.

## INTRODUCTION

Autosomal dominant polycystic kidney disease (ADPKD) is the most common hereditary kidney disease (Cornec-Le Gall et al., 2019) and characterised by fluid-filled renal cysts that expand throughout a patient's lifespan. This is associated with inflammation and fibrosis, resulting in replacement of the healthy kidney parenchyma by cystic tissue and frequently leading to kidney excretory failure. Most cases of ADPKD are associated with pathogenic variants of *PKD1* or *PKD2* (Lanktree et al., 2018), encoding polycystin proteins that are enriched in primary cilia (Ta et al., 2020). Given that polycystins are detected in kidney tubular epithelium (Ibraghimov-Beskrovnaya et al., 1997; Nauli et al., 2003), and cysts are epithelial in origin, our pathophysiological understanding of ADPKD has historically focused towards cellular and molecular alterations to epithelial cells. Pathogenic variants *PKD1* or *PKD2* alter renal tubular mechanosensation and disrupt intracellular calcium homeostasis (Nauli et al., 2003, 2006). This results in increased levels of cyclic adenosine monophosphate (cAMP) (Scholz et al., 2022; Torres and Harris, 2014), dysregulated epithelial cell proliferation (Nishio et al., 2005; Yamaguchi et al., 2000), ion secretion (Terryn et al., 2011) and cyst formation. Current drugs for ADPKD are thus directed towards the kidney epithelium. The clinically licensed drug, tolvaptan, targets tubular arginine vasopressin-mediated cAMP (Torres et al., 2012), whereas somatostatin mediates inhibition of epithelial chloride transport (Caroli et al., 2013). Other interventions within the translational pipeline target epithelial cell metabolism (Menezes and Germino, 2019). Despite these pharmacological approaches, many patients still progress to kidney failure (Cornec-Le Gall et al., 2019), and so alternative targets that could complement available epithelial-targeted drugs are needed.

Mounting evidence, however, suggests that non-epithelial cell types in the microenvironment surrounding cysts are additional important players in the pathology of ADPKD (Cruz et al., 2017; Dwivedi et al., 2023; Fragiadaki et al., 2020; Zimmerman et al., 2020). Of these, the microvasculature could represent a potential therapeutic target (Perretta-Tejedor et al., 2020). The blood microvasculature, composed of endothelial cells (ECs), performs canonical functions, including tissue oxygenation and nutrient delivery. However, in the kidney, the microvasculature is molecularly and structurally specialised to meet the diverse physiological demands of the organ, including ultrafiltration and solute reabsorption (Dumas et al., 2021). There are several lines of evidence implicating the vasculature in ADPKD. Patients with ADPKD have vascular malformations, including aneurysms, in different organs (Perrone et al., 2015). Indeed, polycystins are expressed in blood vessels, and targeted vascular deletion results in abnormal migration and patterning of the systemic vasculature in mouse embryos (Coxam et al., 2014; Kim et al., 2000; Outeda et al., 2014). Moreover, deleting endothelial polycystins in adult mice alters systemic vascular resistance and blood pressure (Hamzaoui et al., 2022; MacKay et al., 2020). These findings indicate that polycystin loss of function has specific consequences on ECs and the systemic vasculature.

How the specialised vasculature of the kidney is affected by ADPKD is not fully understood. In human kidneys with end-stage ADPKD, angiography, immunostaining and corrosion casting have all demonstrated the presence of extensive, tortuous and disorganised blood vascular networks within the tissue microenvironment surrounding cysts (Bello-Reuss et al., 2001; Wei et al., 2006), findings that have been mirrored in rodent models (Huang et al., 2016; Ogunlade et al., 2018; Xu et al., 2013). Such kidney microvascular alterations could occur in late disease from compression by expanding, fluid-filled cysts. An alternative hypothesis is that vascular alterations in ADPKD occur early, alongside cyst growth, and modify disease severity (Huang et al., 2013; Perretta-Tejedor et al., 2020). For example, peritubular capillary density increases early in mouse kidneys with rapidly progressive ADPKD (Huang et al., 2016). However, the nature of these structural alterations is unclear, and the molecular and functional consequences of ADPKD on the kidney microvasculature are unknown. The timing of such changes in relation to the progression of cystic disease is also unclear.

Here, we harnessed state-of-the-art, complementary techniques to provide insights into the kidney microvasculature in ADPKD. We first built a molecular atlas of the human renal microvasculature in normal and diseased kidneys utilising single-cell transcriptomic datasets, leveraging this to annotate data from kidney tissues obtained from ADPKD individuals with advanced cystic pathology and excretory kidney failure. By utilising mice carrying a hypomorphic *Pkd1* p.R3277C point mutation (RC) identified in patients (Hopp et al., 2012; Sieben and Harris, 2023) in homozygosity (*Pkd1^RC/RC^*), we were able to study earlier disease, including an intermediate postnatal stage and an early embryonic stage of disease progression. Supplementing our molecular insights, we derived structural information on renal microvascular architecture using a novel analytical framework, including three-dimensional (3D) confocal imaging of immunolabelled mouse kidneys, followed by geometric, topological and fractal analyses of the microvasculature. Finally, functional information on kidney vascular perfusion was ascertained using multi-modal magnetic resonance imaging (MRI). Leveraging these approaches, we identified a molecularly distinct subpopulation of blood microvasculature in human ADPKD that deviates from the EC injury signature observed in other kidney diseases. Next, *Pkd1* mutant mouse kidneys were examined postnatally when cystic pathology is well established but before excretory failure. An aberrant endothelial subpopulation was also detected, concurrent with reduced cortical blood perfusion. Strikingly, a disorganised, non-uniform kidney cortical microvasculature was also present when tubules begin to dilate in fetal *Pkd1* mutant mouse kidneys. These findings, harmonised between mouse and human cystic kidney disease, highlight molecular and structural features of the aberrant kidney vasculature in ADPKD. Together, these data support the idea that targeting the vasculature early during disease is a putative strategy to ameliorate the progression of ADPKD.

## RESULTS

### Single-cell transcriptomic meta-analysis of human kidney vasculature

**Fig. 1.**
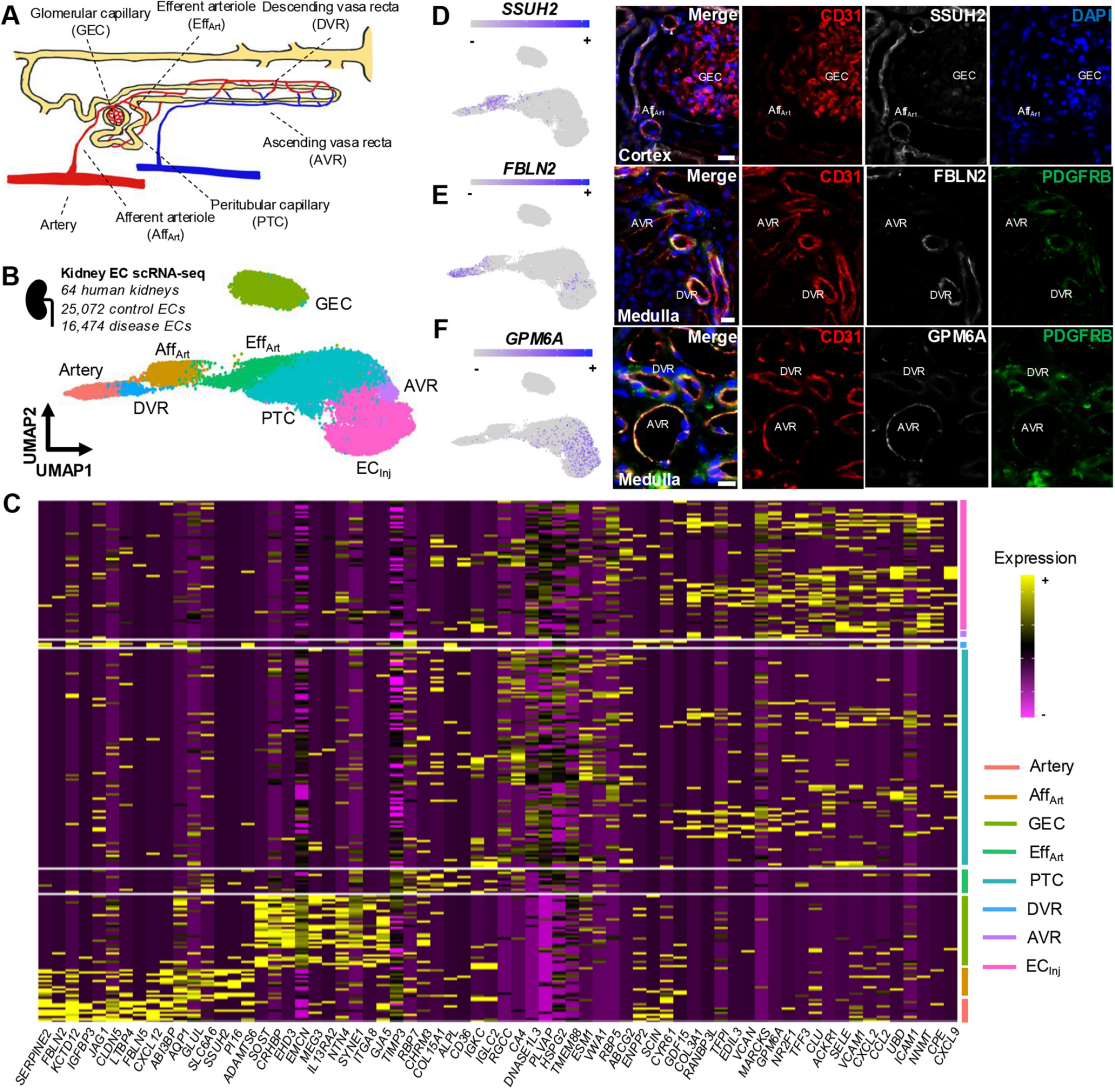
**A single-cell RNA-sequencing census of blood vasculature in the human kidney.** (A) Schematic of kidney blood vascular subsets including arteries, afferent (Aff_Art_) and efferent (Eff_Art_) arterioles, glomerular endothelial cells (GEC), peritubular capillaries (PTC), ascending vasa recta (AVR) and descending vasa recta (DVR). The nephron (yellow) is shown for spatial orientation. (B) Uniform manifold approximation and projection (UMAP) of 41,546 blood endothelial cells (ECs) from 64 human kidneys using publicly available single-cell RNA sequencing (scRNA-seq) data. Seven clusters correspond to annotations in A, with an additional inflammation-activated EC (EC_Inj_) cluster. (C) Heatmap showing ten differentially expressed genes (DEGs) per cluster, with high (yellow) and low (purple) expression coloured. Cluster territories are indicated on the right. (D-F) UMAP and immunofluorescence validation of vascular markers. (D) SSUH2 is a marker of Aff_Art_ (log_2_FC=2.19, *P*<0.0001) and expressed in CD31+ vessels adjacent to glomeruli, containing GEC. (E) FBLN2 is a marker of DVR (log_2_FC=1.55, *P*<0.0001), expressed in CD31^+^ medullary vessels with PDGFRβ^+^ pericyte coverage. (F) GPM6A is a marker of AVR (log_2_FC=1.24, *P*=1.17×10^−68^), expressed in CD31^+^ medullary vessels without PDGFRβ+ pericyte coverage. Images represent five slides across *n*=3 human kidneys. Scale bars: 50 μm.

Before defining how the vasculature is altered in ADPKD, we sought to generate a reference atlas consisting of the transcriptional signatures of each segment of the human kidney vasculature. The kidney is perfused by a hierarchically arranged system of arterial vessels and arterioles. In the cortex, ultrafiltration is performed by glomerular vasculature and solute reabsorption by peritubular capillaries, whereas in the medulla, urinary concentration occurs by solute exchange between vasa recta and specialised tubular epithelium (Fig. 1A). To transcriptionally resolve all these subsets and define a census of EC molecular markers that could be applied to any kidney single-cell transcriptomic dataset, we performed a meta-analysis of existing single-cell RNA-sequencing (scRNA-seq) data, featuring 41,546 blood ECs, defined by co-expression of pan-endothelial markers cadherin (*CDH*)*5* and vascular endothelial growth factor receptor (*VEGFR*)*1* (also known as *FLT1*), pooled across a total of 64 human kidneys (Fig. S1A) (Jafree et al., 2022 preprint). These included 25,072 ECs from healthy kidneys and 16,474 ECs from kidneys with different aetiologies of chronic kidney disease (Kuppe et al., 2021) or chronic transplant rejection (Jafree et al., 2022 preprint; Malone et al., 2020).

We transcriptionally resolved eight distinct clusters of blood ECs (Fig. 1B). Computation of differentially expressed genes (DEGs) (Fig. 1C) provided lists of molecular markers (Table S1) that were used to annotate each cluster, informed by previous studies molecularly characterising kidney EC subsets in mouse (Barry et al., 2019; Dumas et al., 2020) and human (Lake et al., 2019). Seven of the eight clusters corresponded to anatomically distinct segments of the kidney blood vasculature. Glomerular capillaries clustered separately from the remainder of ECs, expressing established markers including EH domain containing 3 (*EHD3*) (Barry et al., 2019; Chung et al., 2020; Miao et al., 2021). Another EC cluster was enriched for the arterial elastic lamina extracellular matrix components fibulin (*FBLN*)*2* and *FBLN5* (Chapman et al., 2010), alongside the arterial endothelium-enriched chemokine C-X-C motif chemokine ligand (*CXCL*)*12* (Takabatake et al., 2009), consistent with an arterial identity. The remainder of the EC clusters did not have as clear transcriptional distinction, and no individual marker was completely specific for any given EC cluster detected in our analysis. Afferent arterioles were classified based on previous studies that identified their ECs to express peptidase inhibitor 16 (*PI16*) (Dumas et al., 2020) and solute carrier family (SLC) 6 member 6 (*SLC6A6*) (Barry et al., 2019). ECs expressing not only arterial markers such as *CXCL12*, but also plasmalemma vesicle associated protein (*PLVAP*) (Lake et al., 2019) and tissue inhibitor of matrix metalloproteinase 3 (*TIMP3*) (Macgregor et al., 2009), were classed as efferent arteriole. Peritubular capillaries also expressed *PLVAP* (Barry et al., 2019; Dumas et al., 2020), while concurrently expressing carbonic anhydrase 4 (*CA4*) (Lönnerholm and Wistrand, 1991). With regard to the medullary segments of the vasculature, the descending vasa recta shared arterial markers such as *FBLN2* (Lake et al., 2019), while also expressing solute carrier family 14 member 1 (*SLC14A1*) (Dumas et al., 2020), whereas the ascending vasa recta were characterised based on the presence of insulin-like growth factor binding protein (*IGFBP*)7, with scant *IGFBP5* transcripts detected (Barry et al., 2019). We further observed a cluster that was unique to the 16,474 ECs from diseased kidneys and was enriched for transcripts associated with inflammatory-mediated activation of ECs, such as inflammatory cell adhesion molecule 1 (*ICAM1*) and vascular cell adhesion molecule 1 (*VCAM1*) (Oates et al., 2022). As these *VCAM1^+^ ICAM1^+^* ECs were clustered together irrespective of the disease they came from, they likely represent a common transcriptional signature of kidney endothelial injury, and we therefore labelled them as ‘injured’ endothelial cells (EC_Inj_).

To validate our annotations, we assessed the expression of novel marker genes within selected EC populations not previously reported in the literature and validated these at the protein level, using immunofluorescence of tissue sections from human kidney. We selected three genes based on their high differential expression with log_2_ fold change (FC) value>1, their specificity for ECs as opposed to other non-EC cell types within the kidney dataset (Fig. S1B), and their commonality across multiple datasets and donors in the meta-analysis (Fig. S1C). One of the top candidates identified in the afferent arteriolar cluster was ssu-2 homolog (*SSUH2*; log_2_FC=2.19, adjusted *P*<0.0001), a transcriptional regulator associated with odontogenesis (Xiong et al., 2017). Correspondingly, SSUH2 was detected in large-diameter cortical CD31^+^ (also known as PECAM1^+^) vessels adjacent to glomeruli of human kidneys (Fig. 1D). Conversely, *FBLN2*, which is expressed by large arteries in our dataset, was also enriched in descending vasa recta ECs (log_2_FC=1.55, adjusted *P*<0.0001), whereas ascending vasa recta ECs were enriched for expression of glycoprotein M6A (*GPM6A*; log_2_FC=1.24, adjusted *P*=1.17×10^−68^). We validated these findings using immunofluorescence, utilising platelet derived growth factor receptor beta (PDGRFβ) staining to discriminate mural cells of CD31^+^ ascending vasa recta. Accordingly, FBLN2 was co-expressed by descending vasa recta (Fig. 1E), whereas GPM6A was detected in CD31^+^ medullary vessels without PDGRFβ^+^ mural cell coverage – the ascending vasa recta (Fig. 1F). Thus, by transcriptionally resolving blood EC subsets in the kidney, we have identified and validated novel molecular markers that could serve to reproducibly annotate other single-cell transcriptomic datasets.

### A distinct blood microvascular phenotype in human end-stage ADPKD

**Fig. 2.**
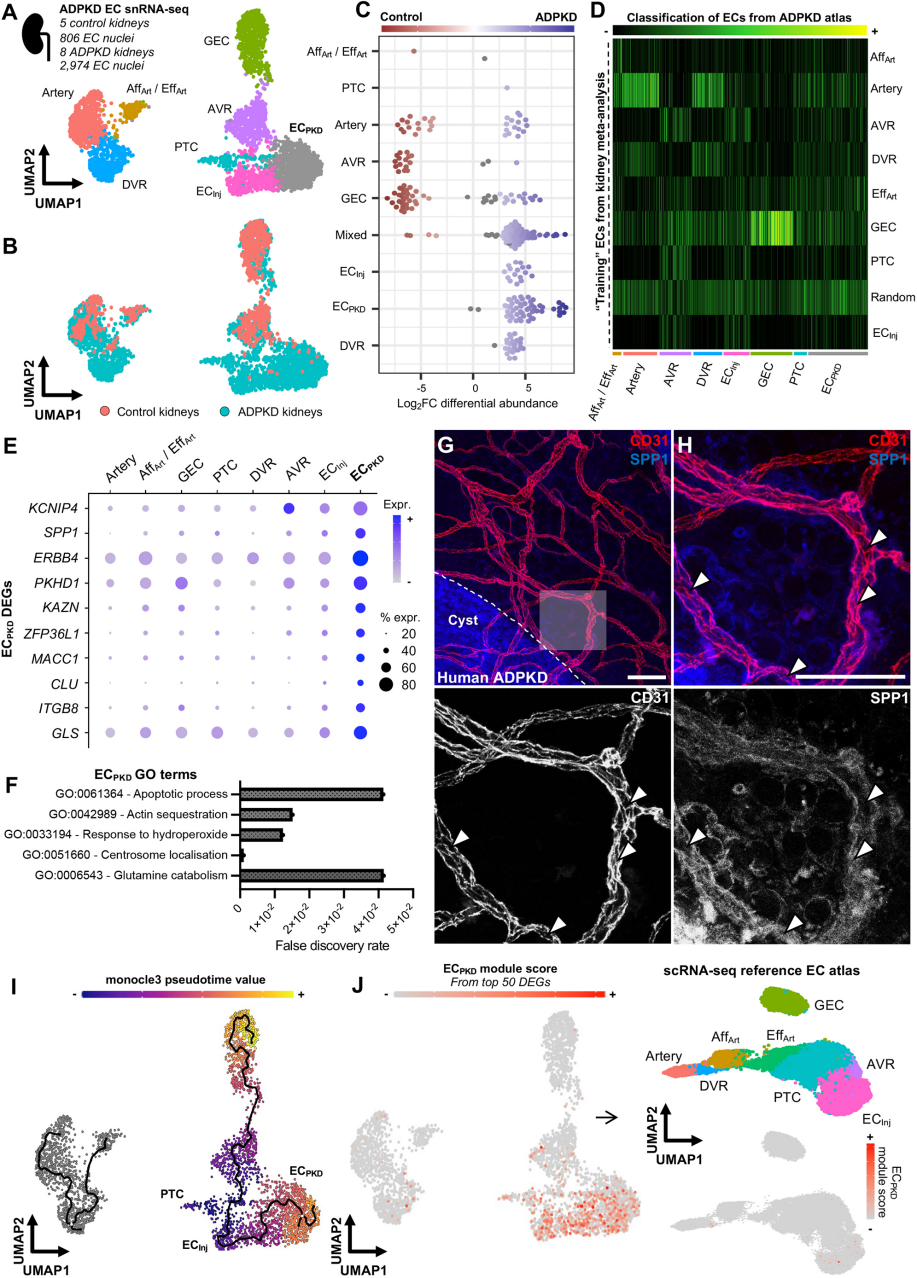
**Single-nucleus RNA sequencing of human end-stage autosomal dominant polycystic kidney disease demonstrates a molecularly distinct kidney blood endothelial subpopulation in cystic kidneys.** (A) UMAP of single-nucleus RNA-sequencing (snRNA-seq) data derived from 3780 blood EC nuclei from five control and eight autosomal dominant polycystic kidney disease (ADPKD) kidney tissues, revealing eight transcriptionally distinct clusters, including a unique cluster (grey) termed EC_PKD_. (B) UMAP from A, grouped by sample to illustrate cell-type abundance. (C) Differential abundance analysis (computed using miloR), showing distributions of each cellular ‘neighbourhood’ within the samples and overabundance of EC_Inj_, EC_PKD_ and DVR in ADPKD. (D) Random forest classification (computed using SingleCellNet) comparing ADPKD kidney ECs to a scRNA-seq reference (Fig. 1), with heatmap intensity indicating transcriptional similarity. (E) Dot plot of the top DEGs in EC_PKD_, with dot size representing the proportion of expressing cells and colour intensity indicating expression level. (F) Gene Ontology (GO) analysis of EC_PKD_ DEGs, with GO terms (*y*-axis) and false discovery rate (FDR; *x*-axis). (G) Confocal microscopy of ADPKD kidney tissues stained for CD31 and SPP1. Dashed line marks SPP1^+^ cyst epithelium; the transparent box highlights the region shown in H. Scale bar: 100 μm. (H) High-magnification image from G, showing SPP1 expression in CD31^+^ vasculature (arrowheads). Single-colour panels for CD31 (bottom left) and SPP1 (bottom right) are also shown. Scale bar: 100 μm. (I) Pseudotime trajectory analysis performed using the monocle3 package in R. The UMAP is identical to that shown in A and B. The overlaid trajectory line represents the predicted cellular progression from PTC through EC_Inj_ to EC_PKD_. Cells are coloured by pseudotime, with purple indicating the root (earliest state) and yellow marking the terminal state. (J) Analysis of EC_PKD_ module score expression in the snRNA-seq and scRNA-seq EC atlases. Using the snRNA-seq ADPKD EC atlas, the top 50 DEGs of EC_PKD_ were used to calculate a module score in Seurat (left). The expression of this module score was then assessed within the scRNA-seq kidney EC dataset (top right), with scant expression across EC cells in this dataset (bottom right).

Having molecularly defined the known subsets of blood ECs within the human kidney, we applied these findings to re-annotate single-nucleus RNA-sequencing (snRNA-seq) data of the kidney ECs in human ADPKD. Data from a recent snRNA-seq study (Muto et al., 2022) were analysed, comprising data from five control kidneys (*n=*806 ECs) and eight kidneys with ADPKD harvested from individuals with severe kidney excretory failure (*n=*2974 ECs), totalling 3780 EC nuclei (Fig. S2A). The nuclei resolved into eight transcriptionally distinct clusters (Fig. 2A), with DEGs for each cluster shown in Table S2. These clusters included all the subsets derived from our earlier single-cell transcriptome meta-analysis, including *FBLN2*^+^ arteries and descending vasa recta, a hybrid afferent/efferent arteriolar cluster enriched for *SSUH2*, and *GPM6A^+^* ascending vasa recta (Fig. S2B). Glomerular or arterial ECs, efferent and afferent arterioles and ascending vasa recta were detected in both control and ADPKD kidneys. Conversely, by visual inspection of the uniform manifold approximation and projection (UMAP) (Fig. 2B) and quantification of cell types in each dataset (Fig. S2C), and through differential abundance analysis (Dann et al., 2022) (Fig. 2C), we detected an uneven distribution of other EC types in ADPKD compared with normal kidneys. Descending vasa recta and peritubular capillaries were predominantly detected in ADPKD kidneys, likely to be a combination of sampling bias, namely the relatively low number of ECs sampled in healthy kidneys and the depletion of peritubular capillary ECs by snRNA-seq (Lake et al., 2019). We resolved two further EC subsets enriched in ADPKD kidneys. Akin to our scRNA-seq meta-analysis, which identified a common kidney endothelial injury signature, one cluster was enriched for markers of EC injury or adhesion, including *ICAM1* and *VCAM1*. However, the second subset did not map to any cluster detected in our earlier meta-analysis. To verify this in an unbiased fashion, we trained a random forest classifier (Tan and Cahan, 2019) using the EC annotations in the meta-analysis to assess ADPKD EC subtypes captured in the snRNA-seq. This analysis is shown in a classification heatmap, in which each bar represents a classification score, indicating similarity between cell types (Fig. 2D). We identified similarity between common EC subtypes in the ADPKD scRNA-seq atlas compared to the previous scRNA-seq reference, including glomerular endothelial cells (GECs), arterial ECs, afferent and efferent arteriolar ECs, and ascending or descending vasa recta. Some transcripts were differentially expressed between classical kidney EC subsets in control and ADPKD tissues (Fig. S2D). EC_Inj_ also shared transcriptionally similar signatures between the meta-analysis and the ADPKD data. The unknown subset of ADPKD-enriched ECs, however, was not transcriptionally similar to any kidney EC subset in health or diseased samples. Given the exclusive presence of these unknown ECs in ADPKD kidneys, and their distinct transcriptional profile from the common *ICAM1^+^ VCAM1^+^* EC injury signature, we referred to this unique cluster of cells as EC_PKD_.

To interrogate the molecular phenotype of EC_PKD_ more closely, we inspected the top DEGs for this cluster compared with all other clusters in the ADPKD snRNA-seq dataset (Fig. 2E). Several identified DEGs have plausible relationships to ADPKD pathogenesis and progression, with the top three candidates by adjusted *P-*value discussed below. Kv channel-interacting protein 4 (*KCNIP4*) – a potassium channel-interacting protein that responds to intracellular calcium levels (Pruunsild and Timmusk, 2005), known to be impaired in *Pkd1*-deficient ECs (Nauli et al., 2008) – was enriched in EC_PKD_ (log_2_FC=0.71, adjusted *P*=1.4×10^−70^). The inflammatory mediator (Trostel et al., 2018) and renal disease biomarker (Khamissi et al., 2022; Kim et al., 2022; Steinbrenner et al., 2023) osteopontin (*SPP1*) was enriched in EC_PKD_ (log_2_FC=1.22, adjusted *P*=6.26×10^−63^), as was Erb-B2 receptor tyrosine kinase 4 (*ERBB4*; log_2_FC=0.83, adjusted *P*=1.7×10^−58^), the expression of which modulates cyst growth in mice and human cyst epithelial cell lines (Streets et al., 2017; Zeng et al., 2014). EC_PKD_ was also enriched for *PKHD1*, which encodes fibrocystin, interacts with *PKD1* (Olson et al., 2019) and is associated with autosomal recessive polycystic kidney disease (PKD) (log_2_FC=0.96, adjusted *P*=1.1×10^−56^), although *PKD1* was not within the list of DEGs. Hypoxia inducible factor (*HIF*)*1A* (log_2_FC=0.48, adjusted *P*=3.0×10^−13^) was also enriched in EC_PKD_, which was noteworthy, given previous reports of regional hypoxia in PKD driving HIF accumulation postulated to contribute to hypervascularity within cystic kidneys (Bernhardt et al., 2007). We then performed Gene Ontology (GO) analysis of the DEGs identified in the EC_PKD_ subset (Fig. 2F). EC_PKD_ was enriched for pathways involved in glutamine catabolism [FC=63.3, false discovery rate (FDR)=4.2×10^−2^], apoptosis (FC=63.3, FDR=4.1×10^−2^) and GO terms relating to polarity, including centrosome localisation (FC=38.0, FDR=1.1×10^−3^) and actin sequestration (FC=28.5, FDR=1.5×10^−2^). Confirming the uniqueness of the EC_PKD_ phenotype to ADPKD, we found that the top DEGs enriched in EC_PKD_ were detected at scant levels, or were not expressed, in our scRNA-seq reference atlas of human healthy and diseased kidney microvascular ECs (Fig. S2E), with the exception of the RNA-binding protein butyrate response factor 1 (ZFP36L1), which is required for normal vascularisation (Bell et al., 2006). We further assessed genes expressed by EC_PKD_ at lower levels on average compared to other cell types within the snRNA-seq dataset (Table S3). EC_PKD_ had significantly lower expression of vascular tyrosine kinases essential for normal vascular development and angiogenesis (He et al., 2019; Perretta-Tejedor et al., 2020) (Fig. S2F). These include *VEGFR1* (log_2_FC=−0.72, adjusted *P*=4.63×10^−21^) and *VEGFR2* (also known as *KDR*) (log_2_FC=−0.53, adjusted *P*=4.01×10^−10^), but not *VEGFR3* (also known as *FLT4*) (adjusted *P*>0.05), tyrosine kinase with immunoglobulin-like and EGF-like domains (*TIE*)*2* (also known as *TEK*) (log_2_FC=−0.81, adjusted *P*=1.03×10^−30^) and, to a lesser degree, *TIE1* (log_2_FC=−0.26, adjusted *P*=1.53×10^−6^). Altogether, these molecular analyses suggest that EC_PKD_ possesses a transcriptome associated with abnormal metabolism and impaired angiogenesis.

We then sought to validate the presence of this transcriptionally distinct EC cluster *in vivo*. Fresh cystic kidney tissue pieces of ∼2 mm^3^ volume were obtained after total nephrectomy from two individuals with late-stage ADPKD, with minimal residual normal kidney tissue. We applied wholemount immunolabelling for the blood vascular marker CD31, optical clearing and confocal 3D imaging (Jafree et al., 2019, 2020). From the markers of EC_PKD_ within the snRNA-seq data, we selected *SPP1* as a candidate, given that it was the top DEG encoding a secreted protein not previously known to be expressed by ECs in this context (Kim et al., 2022). Upon 3D imaging of optically cleared ADPKD tissue, we observed networks of CD31^+^ blood vasculature near SPP1^+^ cyst-lining cells (Fig. 2G). Upon higher magnification, we observed co-expression of CD31 and SPP1 within the microvasculature (Fig. 2H). As evident from our imaging data, however, SPP1 expression within the kidney is not unique to the microvasculature. We therefore explored non-endothelial expression of *SPP1* within the human ADPKD snRNA-seq dataset and found that it was significantly upregulated in multiple nephron epithelial populations, fibroblasts and immune cells (Fig. S2G). Interestingly, *SPP1* expression was not significantly upregulated when the endothelial clusters were pooled together (log_2_FC=0.31, adjusted *P*>0.05), highlighting the importance of subclustering and annotation to resolve kidney microvascular heterogeneity.

Given that the canonical marker genes for kidney microvascular segments were not expressed in EC_PKD_ (Fig. S2B), we further interrogated our snRNA-seq data to give clues as to the identity of this subset. Trajectory inference of our ADPKD EC snRNA-seq dataset was performed using monocle3 (Cao et al., 2019), before applying pseudotime analysis, where the UMAP was colour coded according to its pseudotime from the initiation of the trajectory. Our analyses predicted a trajectory beginning at peritubular capillary endothelium and transitioning through *ICAM1^+^ VCAM1^+^* injured ECs before terminating at EC_PKD_ (Fig. 2I), implying that injured peritubular capillaries potentially give rise to EC_PKD_ in human ADPKD. To verify the uniqueness of this subset for ADPKD, we computed a module score for EC_PKD_ by binning the average expression of the top 50 DEGs for EC_PKD_ and subtracting the aggregated expression of randomly selected control genes across cells (Hao et al., 2024). The EC_PKD_ module score was applied to our reference scRNA-seq atlas of kidney microvasculature, featuring diseased samples including those from individuals with transplant failure and chronic kidney disease (Jafree et al., 2022 preprint). We found that few cells were enriched for the EC_PKD_ module score within our reference scRNA-seq atlas (Fig. 2J), suggesting that the identified population of endothelium is unique to human ADPKD.

### EC_PKD_ are detected in a mouse model of ADPKD

In human ADPKD, kidneys tend to be removed in advanced stages of the disease, precluding the analysis of earlier timepoints in the disease process. We therefore turned to *Pkd1^RC/RC^* mice, a representative animal model that mimics the slow temporal progression of human ADPKD (Hopp et al., 2012; Sieben and Harris, 2023). This would enable us to test the hypothesis that EC_PKD_ were present at a postnatal stage, when cystic pathology is well established but before kidney excretory functional failure. We also examined mutant mouse kidneys in the fetal period when tubules are beginning to dilate. To mitigate the effect of biological sex on cystic disease progression reported in *Pkd1^RC/RC^* mice (Arroyo et al., 2021), only male mice were used.

**Fig. 3.**
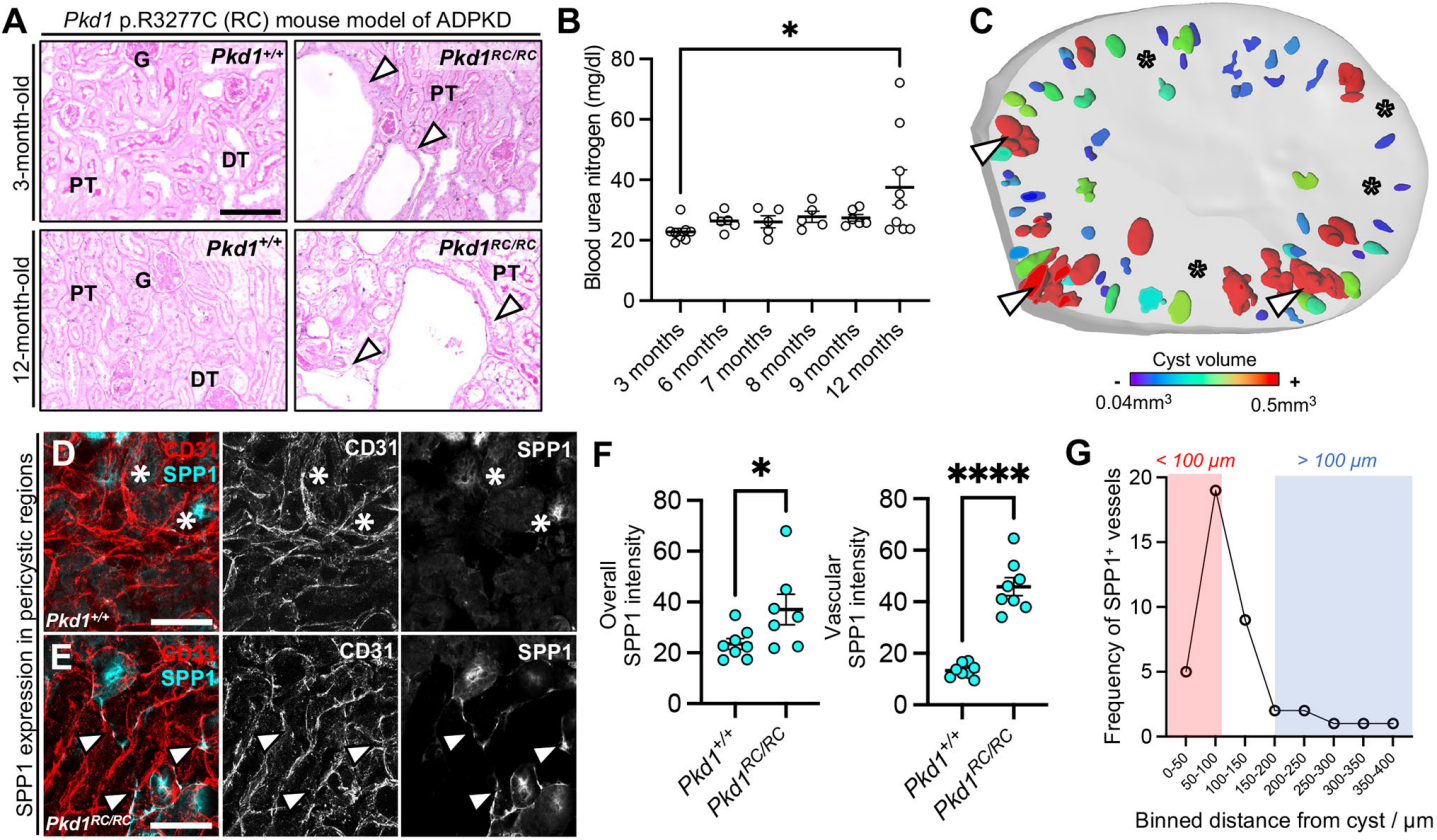
**Molecular changes to blood vasculature in a mouse model of ADPKD at an intermediate disease stage.** (A) Periodic acid–Schiff staining of *Pkd1* p.R3277C (RC) mouse kidneys at 3 and 12 months in wild-type (*Pkd1^+/+^*) or homozygous (*Pkd1^RC/RC^*) littermates. Cysts are indicated with arrowheads. Representative of four slides per kidney (*n=*4 mice per group). Scale bar: 100 μm. DT, distal tubule; G, glomerulus; PT, proximal tubule. (B) Blood urea nitrogen (BUN) levels in *Pkd1^RC/RC^* mice over time. Each point represents an individual mouse. One-way ANOVA showed significant differences (*F*=2.6, *P*=0.049), with Tukey's post hoc test identifying significance between 3 and 12 months (*P*=0.023). (C) 3D rendering of lightsheet microscopy from a 3-month-old *Pkd1^RC/RC^* kidney. Cysts (coloured) are mainly cortical, with size mapped from small (purple, <0.04 mm³) to large (red, >0.5 mm³). Arrowheads indicate cysts; asterisks mark adjacent regions. The analysis was repeated in three kidneys, each from a separate mouse. (D,E) Representative confocal microscopy of *Pkd1^+/+^* (D) and *Pkd1^RC/RC^* (E) kidneys at 3 months, stained for EMCN and SPP1. In D, asterisks indicate nonvascular SPP1 expression; in E, arrowheads mark SPP1^+^ vasculature. Scale bars: 50 µm. (F) Quantification of SPP1 fluorescence from D and E. Left: each point represents a tissue region (*n*=3 per group). Right: vessel-specific analysis, with each point showing mean fluorescence from ten vessel branches. Unpaired two-tailed Student's *t*-test revealed significant differences in overall SPP1 expression (mean difference 13.6±6.0, 95% c.i.=0.66-26.5, *P=*0.041) and vascular SPP1 (mean difference=33.6±3.6, 95% c.i.=24.8-40.3. *P<*0.0001). *P<0.0332, ****P<0.0001.

Histological analysis of *Pkd1^RC/RC^* mouse kidneys demonstrated the presence of epithelial cysts at 3 months of age (Fig. 3A), with a corresponding increase in kidney-to-body weight ratio at this timepoint compared to that in *Pkd1^+/+^* controls (Fig. S3A), an elevation that persisted up to 12 months of age (Fig. S3B). However, measuring blood urea nitrogen (BUN) in *Pkd1^RC/RC^* mice at intervals over 1 year revealed that this surrogate marker of kidney excretory function did not significantly increase until the 12-month timepoint (mean difference=14.8 mg/dl, 95% c.i.=1.4-28.1, *P=*0.023) (Fig. 3B). Using the 488 nm laser of our lightsheet microscope to image autofluorescence and delineate tubular epithelial microstructure, we were able to clearly visualise microscopic cysts in 3-month-old *Pkd1^RC/RC^* kidney vibratome slices, enabling quantification of cystic burden (Fig. 3C). Cyst volumes ranged from below 0.04 mm^3^ to above 0.5 mm^3^, in line with previous two-dimensional characterisation of cyst sizes within this mouse model (Hopp et al., 2012; Sieben and Harris, 2023). Across a total of 166 cysts measured across kidney slices from *n=*3 *Pkd1^RC/RC^* 3-month-old mice, the mean cyst volume was calculated as 3.85×10^−5^±2.93×10^−5^ mm^3^, and we found that 2.32±1.89% of the kidney volume was occupied by cysts in *Pkd1^RC/RC^* mice. This characterisation implies that 3 months is an intermediate timepoint in cystic burden and disease severity in this model, preceding inexorable decline in kidney excretory function observed from 12 months of age.

We then sought to establish whether the EC_PKD_ population detected in the blood microvasculature of human ADPKD kidneys could also be found at the 3-month intermediate stage of disease progression in the *Pkd1^RC/RC^* mouse model. We obtained 500 μm-thick vibratome sections from *Pkd1^+/+^* or *Pkd1^RC/RC^* mouse kidneys, with samples including both cortex and medulla. CD31 was used to immunolabel the vasculature, with co-labelling performed for SPP1, used to demarcate the EC_PKD_ population in our human imaging data. Scant SPP1 expression was detected in CD31^+^ capillary vasculature within *Pkd1^+/+^* kidneys at 3 months (Fig. 3D). Conversely, SPP1^+^ ECs were found interspersed amongst the peritubular capillary network in 3-month-old *Pkd1^RC/RC^* mouse kidneys (Fig. 3E). The fluorescence intensity of SPP1 was quantified from these 3D images, both across the kidney volume and individually within CD31^+^ vasculature (Fig. 3F). When averaged across the kidney volume, the SPP1 fluorescence intensity significantly increased in *Pkd1^RC/RC^* mouse kidneys compared with wild-type control kidneys (mean difference=13.6±6.0, 95% c.i.=0.66-26.5, *P=*0.041). The mean SPP1 fluorescence intensity of CD31^+^ vasculature also increased over threefold in homozygous mice compared to wild-type controls (mean difference=33.6±3.6, 95% c.i.=24.8-40.3, *P<*0.0001). We also analysed the distance of SPP1^+^ CD31^+^ blood vessels relative to the nearest neighbouring cyst epithelial boundary (Fig. 3G). Across *n=*3 *Pkd1^RC/RC^* mice and 40 individual SPP1^+^ CD31^+^ vessels, we found 60% (24 out of 40) of the SPP1^+^ vessels to be within 100 μm from the nearest cyst boundary, whereas the remaining 40% (16 out of 40) were greater than 100 μm away from cysts. Thus, the EC_PKD_ population observed in human kidneys is also found at an intermediate stage of disease in a mouse model of ADPKD.

To explore whether the EC_PKD_ subpopulation occurs independently of abrogated *Pkd1* function in kidney microvasculature, we interrogated kidney snRNA-seq data of an alternative mouse model. In these mice, doxycycline-dependent deletion of *Pkd1* within the *Pax8*^+^ lineage causes progressive cystic disease with doubling of kidney-to-body weight ratio by 4 months of age (Muto et al., 2024). Importantly, *Pax8* lineage recombination in these mice occurs in tubular epithelium, but not in vasculature (Traykova-Brauch et al., 2008), providing a model of cystic kidney disease in which *Pkd1* is not deleted from ECs. The aggregated dataset included male cystic mice and doxycycline-treated littermate controls at early (66 days), moderate (100 days) and advanced (130 days) disease stages (Fig. S4A). The endothelial cluster was extracted from this dataset (Fig. S4B), and subclustering was performed to identify kidney microvascular subsets (Fig. S4C,D). Unlike in our human ADPKD and murine *Pkd1^RC/RC^* datasets, no distinct subcluster resembling EC_PKD_ was identified in these mice, and *Spp1* expression was scant or absent across each kidney microvascular EC subset (Fig. S4E).

### Aberrant microvascular patterning in a mouse model of PKD revealed by geometric, fractal and topological analysis at early and intermediate disease stages

**Fig. 4.**
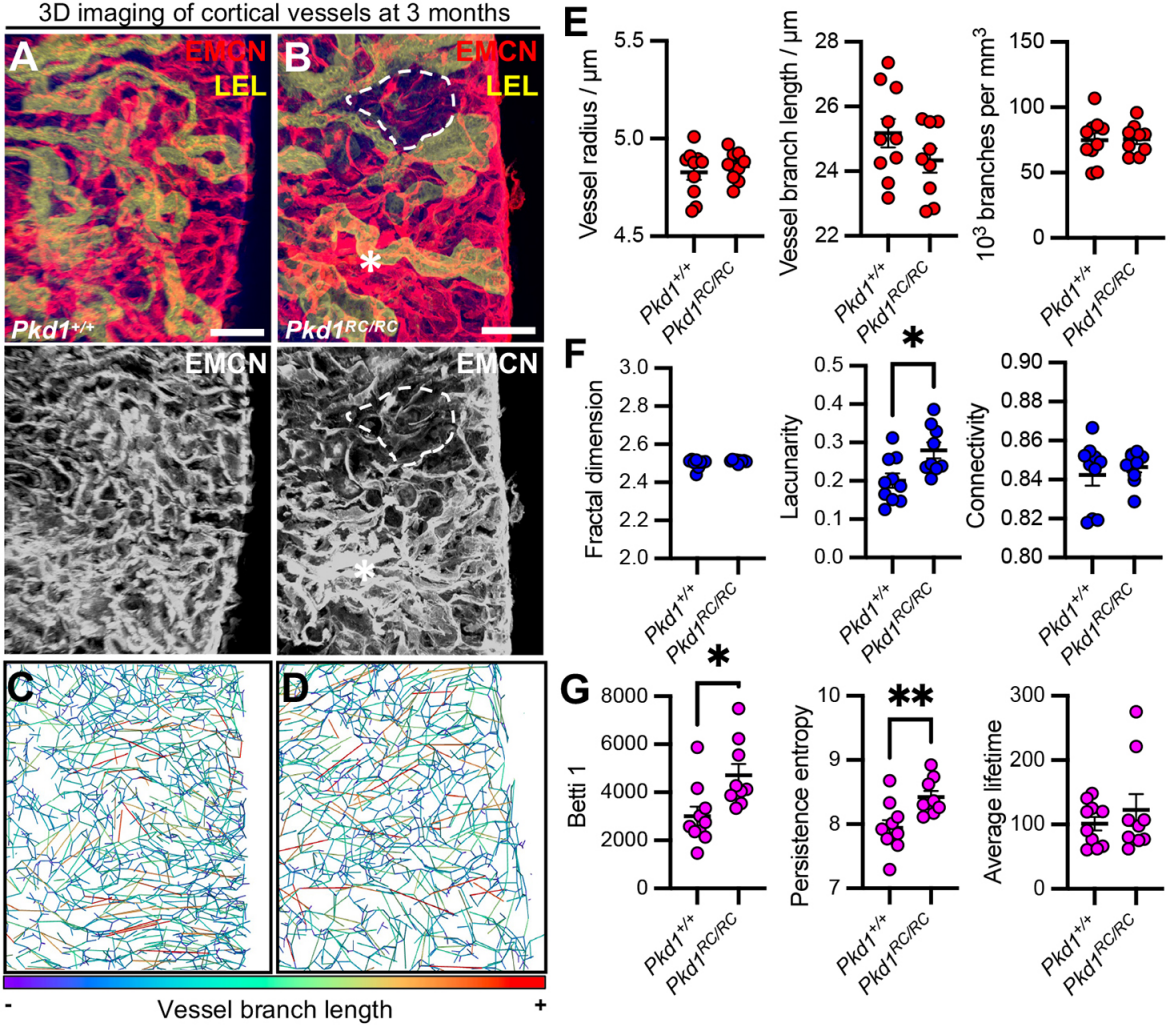
**3D geometric, fractal and topological analysis of the kidney cortical blood microvasculature in a mouse model of ADPKD at an intermediate disease stage.** (A,B) 3D reconstructions of endomucin (EMCN)^+^ vasculature in vibratome slices from *Pkd1^+/+^* (A) and *Pkd1^RC/RC^* (B) kidneys at 3 months of age, stained with Tomato lectin (LEL) to distinguish tubules and cortical–medullary orientation. The *Pkd1^RC/RC^* kidney shows regions of sparse (dashed lines) and dense (asterisks) vascularisation. Scale bars: 50 μm. (C,D) Vessel geometry quantification of EMCN^+^ cortical vasculature using VesselVio in *Pkd1^+/+^* (C) and *Pkd1^RC/RC^* (D) kidneys. Wire skeletons are colour-coded by branch length. (E-G) 3D analysis of the cortical vasculature including geometric analysis (E), with quantification of vessel radius, length and density (vessel branches per mm³). Unpaired two-tailed Student's *t-*test showed no significant differences between groups. (F) Fractal analysis, including vascular structure, including fractal dimension, lacunarity, and connectivity. Lacunarity was significantly different between groups (mean difference=0.079±0.028, 95% c.i.=0.020-0.14, *P=*0.012). (G) Topological analysis of Betti 1, persistence entropy, and average lifetime. *Pkd1^RC/RC^* kidneys showed significant differences in Betti 1 (mean difference=1711±604.3, 95% c.i.=435.9-604.3, *P=*0.012) and persistence entropy (mean difference=0.48±0.15, 95% c.i.=0.16-0.81, *P=*0.006). In A-D, images are representative of *n*=4 kidneys per group at 3 months of age, with ≥3 regions scanned per kidney. In E-G, each point represents a region of interest imaged, pooled across *n*=4 mice per group. *P<0.0332, **P<0.0021.

Having established the presence of EC_PKD_ in human cystic kidneys with advanced disease, and in *Pkd1^RC/RC^* mice at an intermediate stage, we assessed whether structural impairment of the microvasculature precedes decline in excretory function in ADPKD. To do this, we immunostained 3-month-old *Pkd1^RC/RC^* kidney tissues and littermate controls for the blood microvascular marker, endomucin (EMCN). We simultaneously labelled these tissues with *Lycopersicon esculentum* (Tomato) lectin, before high-resolution 3D imaging deep into the tissue using confocal microscopy (Jafree et al., 2020). In our hands, wholemount labelling using Tomato lectin labelled tubular epithelia, enabling clear demarcation of the cortex and medulla using 3D microscopy (Fig. S5A), with cysts demarcated by autofluorescence (Fig. S5B). Maximum-intensity projection of the EMCN^+^ vasculature in the cortex demonstrated regularly patterned peritubular capillaries in *Pkd1^+/+^* kidneys (Fig. 4A). Visually, there was non-uniformity in the patterning of the vasculature in the 3-month-old *Pkd1^RC/RC^* kidney cortex (Fig. 4B). We thus analysed renal blood microvascular geometry utilising a prior framework, by segmenting the intact 3D network, prior to leveraging open source 3D vascular analysis software (Bumgarner and Nelson, 2022). Conventional vessel branch geometric properties were quantitated, including the branch length (mapped and visualised using 3D rendering in Fig. 4C,D), radius and vascular density assessed as the number of branches per mm^3^ of tissue. There were, however, no significant differences in EMCN^+^ microvessel branch radius, length or density between *Pkd1^+/+^* and *Pkd1^RC/RC^* mouse kidneys at 3 months (Fig. 4E).

A limitation of geometric analyses is that they do not consider the patterning of the vascular network *en masse* and thus may not capture visually detectable phenotypes. We therefore turned to a novel approach of analysing the kidney microvasculature in the ADPKD mouse model. We leveraged 3D fractal analysis, which better represents the patterning and complexity of the network in 3D space, compared with individual summary statistics such as mean radius and length. Using 3D imaging of EMCN^+^ microvessels, we were able to quantify metrics such as fractal dimension, representing vascular complexity; lacunarity, representing non-uniformity; and connectivity. This analysis demonstrated a significant increase in the heterogeneity of distribution of the vascular network in *Pkd1^RC/RC^* mouse kidneys compared with wild-type control kidneys at 3 months (mean difference in lacunarity=0.079±0.028, 95% c.i.=0.020-0.14, *P=*0.012), without significant changes in complexity (fractal dimension) or connectivity between conditions (Fig. 4F), agreeing with the visual changes observed in 3D microscopy of a more non-uniform vascular network in mutant mice. To obtain deeper insights, we derived topological measures from segmented images of the EMCN^+^ microvasculature, including Betti numbers, representing the number of vascular loops; persistence entropy, representing vascular disorganisation; and average lifetime, representing persistence of patterns in 3D space. We found both the number of loops (mean difference of Betti 1=1711±604.3, 95% c.i.=435.9-604.3, *P=*0.012) and vascular disorganisation (mean difference of persistence entropy=0.48±0.15, 95% c.i.=0.16-0.81, *P=*0.006) to be significantly greater in *Pkd1^RC/RC^* mouse kidneys compared with wild-type control kidneys at 3 months, with no significant change in the persistence of patterns in 3D space (average lifetime, Fig. 4G). In contrast, our 3D analysis of geometric (Fig. S6A), fractal (Fig. S6B) or topological (Fig. S6C) measures detected no significant changes in the EMCN^+^ medullary vasculature of *Pkd1^+/+^* or *Pkd1^RC/RC^* mouse kidneys. Thus, in this murine model of ADPKD, aberrant kidney microvascular remodelling is present, confined to the kidney cortex, and is concurrent with the EC_PKD_ subpopulation, occurring at an intermediate stage that precedes decline of kidney excretory function.

**Fig. 5.**
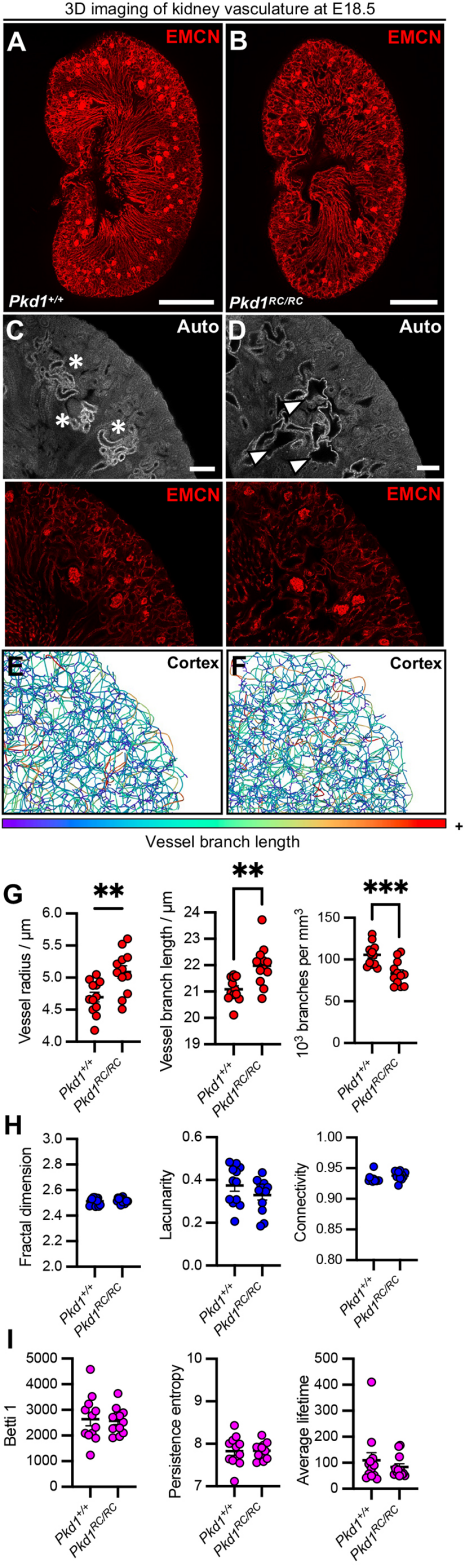
**3D geometric, fractal and topological analysis of the kidney cortical blood microvasculature in a mouse model of ADPKD at a fetal stage.** (A,B) 3D reconstructions of endomucin (EMCN)^+^ vasculature in vibratome slices from *Pkd1^+/+^* (A) and *Pkd1^RC/RC^* (B) kidneys at embryonic day (E)18.5. Scale bars: 500 μm. (C,D) *z-*sections of autofluorescence (top row, grey) and EMCN^+^ vasculature (bottom row, red) from *Pkd1^+/+^* (C) and *Pkd1^RC/RC^* (D) kidney cortex. Autofluorescence of cortical tubular epithelium (asterisks) in *Pkd1^+/+^* kidneys and tubule dilation (arrowheads) in *Pkd1^RC/RC^* kidneys are shown. Scale bars: 100 μm. (E,F) Vessel geometry quantification of EMCN^+^ cortical vasculature using VesselVio in *Pkd1^+/+^* (E) and *Pkd1^RC/RC^* (F) fetal kidneys, with wire skeletons colour-coded by branch length. (G-I) 3D analysis of the cortical vasculature at E18.5, including geometric analysis. (G) *Pkd1^RC/RC^* kidneys showed increased vessel radius (mean difference=0.39±0.12, 95% c.i.=0.14-0.65, *P=*0.004) and branch length (mean difference=0.89±0.26, 95% c.i.=0.36-1.42, *P=*0.002), and a decrease in vascular density (mean difference=22±6, 95% c.i.=10-34, *P=*0.0007). (H) 3D fractal analysis (fractal dimension, lacunarity, connectivity) showed no significant differences between groups. (I) 3D topological analysis (Betti 1, persistence entropy, average lifetime) also showed no significant differences. All images in A-F are representative of *n*=4 kidneys per group at E18.5, and each point in G-I represents an individual region of interest from images pooled across *n*=4 kidneys. **P<0.0021, ***P<0.0002 (unpaired two-tailed Student's *t*-test).

In the *Pkd1^RC/RC^* mouse model, the initiation of cyst formation commences much earlier than postnatal stages, with tubular dilation observed within the late embryonic period (Hopp et al., 2012; Jafree et al., 2019). To profile the kidney blood microvasculature at this early stage, we generated *Pkd1^RC/RC^* mouse embryos and *Pkd1^+/+^* littermate controls at embryonic day (E)18.5. We wholemount stained 200 μm-thick vibratome sections for EMCN to capture microvascular networks from E18.5 *Pkd1^+/+^* (Fig. 5A) or *Pkd1^RC/RC^* (Fig. 5B) kidneys, observing tubular dilation at this prenatal stage (Fig. 5C,D). We also performed segmentation and skeletonisation of the vasculature (Fig. 5E,F) before deriving 3D geometric, fractal and topological metrics. Within the fetal kidney cortex, we observed significant differences in all geometric measures (Fig. 5G), with mean vessel radius (mean difference of radius=0.39±0.12, 95% c.i.=0.14-0.65, *P=*0.004) and branch length (mean difference of branch length=0.89±0.26, 95% c.i.=0.36-1.42, *P=*0.002) increased in mutant kidneys, whereas the vascular density decreased (mean difference in 10^3^ vessels per mm^3^ tissue=22±6, 95% c.i.=10-34, *P=*0.0007). Conversely, no significant differences were observed in fractal (Fig. 5H) or topological (Fig. 5I) analysis of the cortical vasculature at E18.5. Akin to the 3-month timepoint, no significant changes in vascular geometry (Fig. S7A) were found within the E18.5 medullary vasculature of *Pkd1^RC/RC^* kidneys. A small, but significant, decrease was observed in the fractal dimension (Fig. S7B), representing lower vascular complexity, in the medulla of *Pkd1^RC/RC^* embryonic kidneys (mean difference of fractal dimension=0.01±0.0025, 95% c.i.=0.005-0.02, *P=*0.0004). No significant changes in other fractal or topological measures (Fig. S7C) were observed.

### Early reduction in cortical blood flow in a mouse model of PKD

**Fig. 6.**
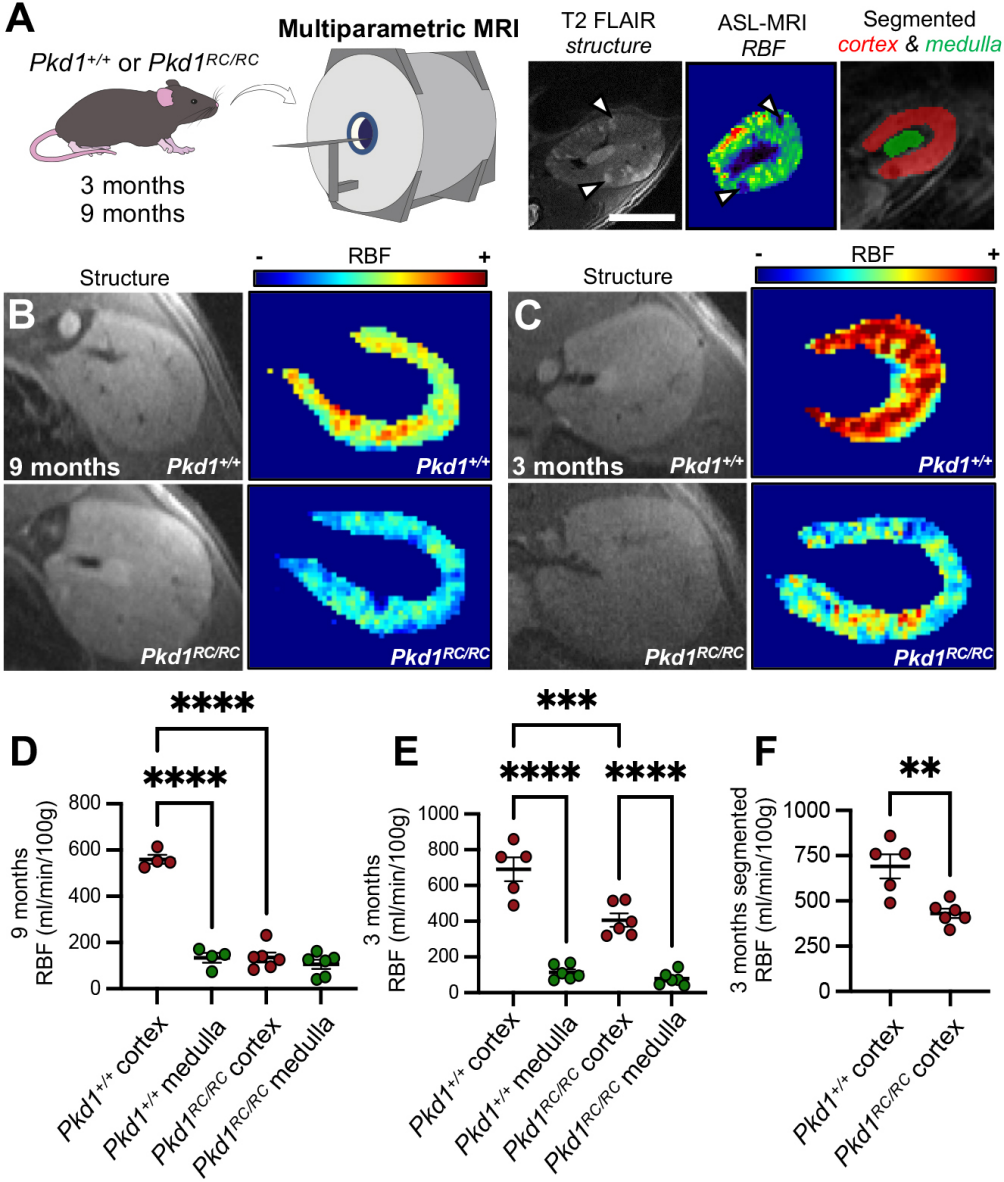
**Multiparametric magnetic resonance imaging shows early regional reduction in cortical blood flow in a mouse model of ADPKD.** (A) Experimental setup. At 9 months or 3 months of age, *Pkd1^+/+^* and *Pkd1^RC/RC^* mice underwent T2-FLAIR magnetic resonance imaging (MRI) for structural imaging and arterial spin labelling (ASL) to assess renal blood flow (RBF), distinguishing cortical and medullary flow. Structural imaging enabled segmentation of cortex from medulla. Arrowheads indicate cysts. Scale bar: 2 mm. (B,C) ASL heatmaps of RBF at 9 months (B) and 3 months (C) show reduced cortical flow in *Pkd1^RC/RC^* kidneys compared to *Pkd1^+/+^* kidneys. (D) Quantification of RBF at 9 months. One-way ANOVA showed significant differences, with reduced RBF in the cortex compared to medulla in wild-type kidneys (mean difference=426.1±31.1 ml/min/100 g, 95% c.i.=331.4-529.8, *P<*0.0001), and a reduction in the cortical RBF in *Pkd1^RC/RC^* compared to wild-type kidneys (mean difference=424.4±30.2 ml/min/100 g, 95% c.i.=337.9-510.0, *P<*0.0001). (E) Quantification of RBF at 3 months. Similar reductions in wild-type kidney RBF between medulla and cortex (mean difference=577.6±53.0 ml/min/100 g, 95% c.i.=428.6-726.6, *P<*0.0001), and between the cortex of *Pkd1^RC/RC^* compared to wild-type kidneys (mean difference=285.0±52.9 ml/min/100 g, 95% c.i.=136.1-434.0, *P=*0.0002), were observed (one-way ANOVA). (F) Quantification of RBF at 3 months, with macroscopic cysts segmented out of *Pkd1^RC/RC^* mouse kidneys. The reduction in cortical RBF was maintained in the *Pkd1^RC/RC^* kidney cortex using this approach (mean difference in 259.6±66.4 ml/min/100 g, 95% c.i.=109.5-409.7, *P=*0.0035; unpaired two-tailed Student's *t*-test). Each data point in D-F represents a kidney measured from an individual mouse, with cortex represented by red points and medulla by green points. **P<0.0021, ***P<0.0002, ****P<0.0001.

Having identified both molecular and structural changes to the vasculature in the *Pkd1^RC/RC^* mouse model of ADPKD, we sought to assess whether these findings had functional correlations, specifically whether blood perfusion within the kidney is altered. To do this, we leveraged a 9.4T MRI scanner to perform *in vivo* imaging of live *Pkd1^+/+^* or *Pkd1^RC/RC^* mice (Fig. 6A). We applied a multimodal contrast-free approach, first assessing the structure of the kidney using a T2-FLAIR sequence, before using arterial spin labelling (ASL) to map and quantify renal blood flow (RBF) (Odudu et al., 2018). As T2-FLAIR was able to discriminate cortex and medulla within the kidney, aligned ASL images from the same mice could be used to discriminate blood flow originating from cortex or medulla. *Pkd1^+/+^* or *Pkd1^RC/RC^* mice were scanned at 3 and 9 months of age, timepoints that precede the significant increase in BUN found in this model.

When visualised using heatmaps, there were striking changes to RBF within the cortex of *Pkd1^RC/RC^* mouse kidneys compared with wild-type kidneys at both 9 months (Fig. 6B) and 3 months (Fig. 6C). We then discerned average values of RBF from each ASL image, analysing cortex and medulla of each kidney separately. There was significantly higher RBF in the cortex compared to the medulla (Fig. 6D) at both 9 months (mean difference=426.1±31.1 ml/min/100 g, 95% c.i.=331.4-529.8, *P<*0.0001) and 3 months (mean difference=577.6±53.0 ml/min/100 g, 95% c.i.=428.6-726.6, *P<*0.0001) in *Pkd1^+/+^* wild-type mouse kidneys, RBF values that corresponded with those reported in the literature (Hueper et al., 2014, 2017; Tewes et al., 2017). We then compared between health and disease, revealing a 76% reduction in mean cortical blood flow at 9 months in *Pkd1^RC/RC^* mouse kidneys compared to wild-type control kidneys (Fig. 6D; mean difference=424.4±30.2 ml/min/100 g, 95% c.i.=337.9-510.0, *P<*0.0001). Strikingly this significant reduction was also observed at the earlier timepoint of 3 months, with a 35% reduction in mean cortical RBF in cystic kidneys compared to wild-type control kidneys (Fig. 6E; mean difference=285.0±52.9 ml/min/100 g, 95% c.i.=136.1-434.0, *P=*0.0002). Medullary blood flow, however, was not significantly different between *Pkd1^RC/RC^* or *Pkd1^+/+^* mouse kidneys at either timepoint. To examine whether the reduction in RBF observed in the cortex of cystic kidneys held after accounting for macroscopic cysts, we paired T2-FLAIR images to segment regions devoid of large cysts visible on the structural MRI sequence (Fig. 6F). Indeed, we found that the significant reduction in RBF observed between *Pkd1^RC/RC^* and *Pkd1^+/+^* kidneys was maintained using this approach (mean difference=259.6±66.4 ml/min/100 g, 95% c.i.=109.5-409.7, *P=*0.0035). The local reduction in blood flow within the cortex of *Pkd1^RC/RC^* mouse kidneys thus corresponds to our molecular and structural data, showing that aberrant vascular remodelling is specific to the cortical microenvironment at an intermediate stage of cystic disease.

## DISCUSSION

This work leveraged a range of complementary and state-of-the-art approaches, including transcriptomics at cellular resolution; 3D imaging of intact, immunolabelled and optically cleared kidney tissues; and *in vivo* imaging of kidney structure and blood perfusion. We began by detecting a transcriptionally distinct subset of kidney blood microvasculature in human individuals with ADPKD, with a molecular profile suggesting abnormal metabolism and impaired angiogenesis, which is not observed in chronic kidney disease or transplant failure. We provide molecular evidence of this perturbed microvascular phenotype at 3 months of age in an orthologous murine model of ADPKD, which was not detectable in an alternative model featuring specific deletion of *Pkd1* in tubular epithelium. At this intermediate stage of disease in the ADPKD mouse model, the perturbed microvascular phenotype was found alongside disorganised and heterogeneous patterning of the microvasculature, which was preceded by changes in vessel geometry before birth. Consistent with these structural aberrations and molecular changes, there was also local impairment of blood flow in the *Pkd1^RC/RC^* mouse kidney cortex at 3 months of age. Collectively, these data support our hypothesis that the kidney blood microvasculature is perturbed at molecular, structural and functional levels in ADPKD. As these perturbations occur prior to the decline in kidney excretory function in the mouse model, our findings implicate the kidney microvasculature as a potential therapeutic target in ADPKD, at stages when kidney regeneration or repair is still plausible.

This study, bridging molecular, structural and functional levels, sheds new lights on the phenotype of the kidney vasculature in ADPKD. Although it has been established that mutations in *Pkd1* cause defects in endothelial development (Coxam et al., 2014; Kim et al., 2000; Outeda et al., 2014) and flow-mediated dilation of the systemic vasculature (Hamzaoui et al., 2022), the phenotype of the kidney microvasculature ADPKD is less well understood, particularly from a molecular standpoint. These experiments address this gap in the literature by interrogating the molecular phenotype of the human and mouse cystic kidney vasculature in depth. By generating and validating a reference scRNA-seq atlas of the kidney microvasculature, applicable to other kidney diseases, we dissected EC subtype-specific transcriptomes from human ADPKD snRNA-seq data (Muto et al., 2022). In so doing, we identified a unique cluster of blood microvascular endothelium present in human ADPKD, which we designate EC_PKD_, that is characterised by altered metabolism and impaired angiogenic signalling. SPP1, which was identified as a molecular marker of EC_PKD_, was detected in the *Pkd1^RC/RC^* mouse model at the 3-month timepoint. However, the lack of evidence for EC_PKD_ within an alternative mouse model, featuring inducible tubular epithelial deletion of *Pkd1* (Muto et al., 2024), implies that this phenotype depends on aberrant *Pkd1* signalling in kidney ECs.

Previous work has shown loss of VEGF–VEGFR signalling as a feature of PKD rodent models (Huang et al., 2016; Raina et al., 2011). However, to our knowledge, our work is the first to demonstrate a molecular consequence of a *Pkd1* mutation on the microvasculature within cystic kidneys. These molecular findings marry with our 3D interrogation of kidney microvascular architecture, which revealed disorganisation, an increased number of loops and heterogeneous patterning at the intermediate stage of 3 months, preceded by abnormal vessel geometry at a fetal timepoint. Our findings reconcile previous angiography, immunohistochemistry and corrosion casting of human end-stage ADPKD explants and mouse models – with some reports indicating abnormal angiogenic remodelling as a feature of ADPKD (Bello-Reuss et al., 2001; Huang et al., 2016; Wei et al., 2006), whereas others suggest a lower overall renal vascular density in cystic disease (Ogunlade et al., 2018; Wei et al., 2006; Xu et al., 2013). Whereas conventional, 3D geometric analysis of the microvasculature failed to find any changes in metrics such as branch radius, length or density at 3 months of age, we developed a more sensitive 3D approach to detect overall patterning metrics of the kidney microvasculature, incorporating topological information and fractal analysis. Interestingly, geometric, but not fractal or topological measures, were altered within E18.5 mutant kidneys. These findings imply that early geometric changes result in a rudimental cortical microvascular network in *Pkd1* mutant kidneys, which then herald defects in vessel patterning and complexity at later, postnatal stages. The temporal evolution of this phenotype could be driven by plasticity during fetal vascularisation, which then declines over the lifespan (Borri et al., 2025). Linking this hypothesis with our bioinformatic analysis, the EC_PKD_ molecular phenotype could represent a maladaptive response to inflammation and injury within the cystic microenvironment. Such structural and molecular changes could thus be responsible for the decline in local cortical perfusion within the kidney, as we demonstrated using ASL of *Pkd1^RC/RC^* mouse kidneys at the 3-month stage of the disease.

This study generates several technical advances, such as our scRNA-seq atlas that, alongside new studies (Zhang et al., 2024), provides a molecular reference for future transcriptomic experiments interrogating human kidney vasculature during health and disease. The novel 3D approach to discern quantifiable fractal and topological information from the kidney vasculature has implications in studying the physiological or pathological vascularisation. This is applicable to clinical contexts relevant to ADPKD, such as intracranial vascular aneurysms in patients, which can lead to life-threatening rupture (Kataoka et al., 2022). The present experiments also have other clinical implications. Specifically, unique molecular changes to the kidney microvasculature were identified in individuals with ADPKD, with structural and functional defects detected at early and intermediate stages of an orthologous mouse model of this disease. Importantly, the changes in *Pkd1^RC/RC^* mouse kidneys were detectable at a timepoint at which ∼98% of the kidney volume remains unoccupied by cysts, with sufficient normal kidney parenchyma to maintain baseline BUN values. As the temporal progression of cystic disease in *Pkd1^RC/RC^* mice is comparable with that in human ADPKD (Hopp et al., 2012), our study hints towards the presence of kidney microvascular abnormalities before the inexorable decline in kidney excretory function observed in patients. Indeed, impaired renal perfusion has been detected in patients with ADPKD using contrast-enhanced magnetic resonance angiography, and flow detected within the renal arteries is an independent predictor of kidney function (King et al., 2003; Torres et al., 2007). The limited resolution of clinically approved *in vivo* imaging makes it challenging to detect regional defects in perfusion; our study addresses this using a non-contrast and higher-resolution approach, demonstrating intrinsic microvascular dysfunction in the *Pkd1^RC/RC^* mouse kidney. It would be useful to apply such techniques to future clinical trials of ADPKD and monitor the response of intrinsic kidney blood perfusion to pharmacological agents. However, MRI has its limitations, including a partial volume effect from averaging RBF measurements with ‘zero’ flow regions caused by large epithelial cysts. Although we have tried to mitigate these in our study by segmenting regions devoid of macroscopic cysts from aligned T2-FLAIR imaging, emerging techniques offer attractive alternatives, such as photoacoustic imaging (Huynh et al., 2024; Ogunlade et al., 2018) or multiphoton microscopy (Molitoris et al., 2022). The resolution capable with these modalities, when paired with the novel 3D analysis described in this paper, could be harnessed to non-invasively assess kidney microvasculature during disease and after treatment. Our findings also have therapeutic implications. It has already been established that manipulation of VEGF–VEGFR signalling alters disease progression in rapidly progressive rodent models of PKD (Huang et al., 2016; Raina et al., 2011; Tao et al., 2007). TIE-mediated angiopoietin signalling, which has been investigated in the context of other kidney diseases (Chang et al., 2022; Dessapt-Baradez et al., 2014; Li et al., 2023b; Long et al., 2008), appeared to be unique to EC_PKD_ and could therefore represent a novel target for microvascular remodelling in ADPKD. This microvascular heterogeneity in ADPKD might be amenable to targeted approaches, such as protein carriers (Engel et al., 2020), nanoparticles (Goodlett et al., 2021) or gene therapy. Such innovative therapeutic approaches could realise the potential of targeting the vasculature in ADPKD while reducing systemic unwanted side-effects.

### Limitations

Despite the detection of structural abnormalities of the kidney microvasculature surrounding cysts in human ADPKD ∼20 years ago (Bello-Reuss et al., 2001; Wei et al., 2006), the well-known clinical association between ADPKD and systemic vascular abnormalities (Perrone et al., 2015), and correlation between RBF and kidney function decline in ADPKD cohort studies (King et al., 2003; Torres et al., 2007), there has been little in the way of application of vascular-based therapies to patients. Here, we provide an in-depth characterisation of the microvasculature in human ADPKD and in a mouse model, although we have not correlated the presence of *PKD1* or *PKD2* pathogenic variants in patients with the microvascular phenotype observed. The uniqueness of the molecular phenotype of the microvasculature observed in ADPKD compared to kidney diseases, and parallel findings in *Pkd1^RC/RC^* mice, could be driven by intrinsic consequences of a *PKD1* pathogenic variant or deletion on endothelial development and function (Hamzaoui et al., 2022; Huang et al., 2016; MacKay et al., 2020). Conversely, microvascular disruption could occur as a result of the surrounding microenvironment, including macrophage-mediated inflammation (Cassini et al., 2018; Li et al., 2023a), fibroblast activation (Dwivedi et al., 2023) or paracrine signalling from cyst epithelium (Kenter et al., 2019). We begin to tease this out in our study by demonstrating the absence of EC_PKD_ in mice with inducible *Pkd1* deletion in *Pax8^+^* tubular cells, which is not reported to target ECs (Traykova-Brauch et al., 2008). For completion, the structural and functional phenotype of the kidney vasculature in this model would need to be examined or, alternatively, mice with endothelial deletion of *Pkd1* would require assessment. We nevertheless demonstrate that kidney microvascular perfusion is impaired from intermediate stages of the disease, providing a cellular target within a therapeutic window during which renal function could still be salvageable. Although our study is descriptive, this work lays an essential groundwork for functional studies towards clinical translation. For example, SPP1 is a key inflammatory player across multiple diseases (Zhao et al., 2024) and has prognostic and therapeutic potential in chronic kidney disease (Ding et al., 2024; Steinbrenner et al., 2023; Stubbs et al., 2022). We identified SPP1 as a marker of EC_PKD_ within our study. However, SPP1 can be expressed by ECs in other contexts, such as in glomerular capillaries during murine and human glomerulonephritis (Fu et al., 2021). Moreover, in humans and mice (Muto et al., 2024) with PKD, SPP1 expression is not confined to the endothelium; therefore, cell type-specific roles warrant further study (Jansson et al., 2024) prior to assessing therapeutic potential. Finally, there are other subsets of endothelium, including venous endothelium and lymphatics, which might be perturbed in ADPKD (Huang et al., 2016; Jafree et al., 2019; Yin et al., 2021) but are not explored in this study owing to their under-representation or absence from scRNA-seq data of the kidney.

### Conclusions

In summary, we resolve the molecular, structural and functional phenotype of the kidney endothelium in ADPKD, manifesting in defective molecular, aberrantly remodelled and poorly functional microvasculature. The presence of this endothelial phenotype precedes inexorable decline in kidney excretory function. Our findings highlight kidney microvascular aberrations as an early hallmark of ADPKD, representing a putative therapeutic target within a timeframe during which the kidney can still be repaired or regenerated. Altogether, the evidence presented in this study strengthens the case for vascular-based therapeutics as an alternative or complementary strategy for ADPKD.

## MATERIALS AND METHODS

### Ethics statement

Ethical approval to obtain ADPKD tissue samples for 3D imaging was granted by the UK National Research Ethics Committee (21/WA/0388) and approved by The Royal Free London NHS Foundation Trust-UCL Biobank Ethical Review Committee (NC.2018.007, B-ERC-RF). All experiments on human kidney tissues were conducted according to the principles expressed in the Declaration of Helsinki. Mouse experiments were carried out according to a UK Home Office project license (PPL: PP1776587) and were compliant with the UK Animals (Scientific Procedures) Act 1986.

### Analysis of scRNA-seq and snRNA-seq data

#### Sample acquisition and pre-processing

We profiled single-cell transcriptomes of the kidney blood microvasculature using previously published scRNA-seq data of healthy and diseased human kidneys. The generation of the dataset has been described previously (Jafree et al., 2022 preprint) and, briefly, includes healthy (donor kidneys not suitable for transplantation, non-rejection biopsies from kidney transplants or non-tumorous regions from tumour nephrectomies) and diseased (chronic kidney disease, transplant failure) samples. Independently, we assessed a previously published snRNA-seq dataset of healthy or ADPKD tissues (Muto et al., 2022) acquired from the Gene Expression Omnibus (GEO) database (GSE185948). Analyses of these two datasets were performed separately in R using Seurat (Hao et al., 2024). Raw count matrices from each dataset were isolated from clusters annotated as ECs, verified by the co-expression of blood EC-enriched genes: *CDH5* and *VEGFR1*. Thereafter, the raw counts were independently log normalised, scaled by expression of all genes detected and subjected to principal component analysis. The number of principal components used for downstream analysis was determined by the point at which principal components cumulatively contributed to 90% of the standard deviation. Integration of cells from each donor was performed using the Harmony package (Korsunsky et al., 2019). Nearest-neighbour graph construction, unsupervised clustering and UMAP were performed using the FindNeighbors, FindClusters and RunUMAP functions, respectively. Any cell clusters recognised as non-blood ECs or low quality and non-specific were removed, and the above procedure was iterated until only blood ECs remained.

#### Cell-type annotation, differential abundance and unbiased cross-dataset comparison

For scRNA-seq and snRNA-seq data, lists of DEGs discriminating between blood EC clusters were generated. These lists were manually inspected and compared with previously published datasets identifying specialised subsets of vasculature within the mouse (Barry et al., 2019; Dumas et al., 2020) and human (Lake et al., 2019) kidney to annotate specialised renal blood EC subtypes. To assess the comparative abundance of cell types within snRNA-seq data, we performed differential abundance testing using miloR (Dann et al., 2022), visualising differences in log_2_FC abundance of each cell type between healthy kidneys and those with ADPKD using a beeswarm plot. To compare cell-type annotations between scRNA-seq and snRNA-seq data, the SingleCellNet (Tan and Cahan, 2019) tool was used, setting scRNA-seq annotations as ‘reference’ and snRNA-seq annotations as ‘query’, and visually compared using a heatmap.

#### Computing DEGs, GO, marker selection and module score

DEGs between distinct blood vascular subsets, or between healthy and ADPKD kidneys, were computed using the FindAllMarkers function, considering genes with an adjusted *P-*value<0.05 or log_2_FC value greater than 0.25. DEGs between healthy and ADPKD kidneys for each blood EC subset were visualised using the EnhancedVolcano package. GO analysis was performed using the PANTHER webtool for gene classification (Mi et al., 2021) as previously described (Jafree et al., 2022 preprint). Marker genes were selected based on the following criteria: (1) an adjusted *P-*value>0.05 from a Wilcoxon rank sum test; (2) a log_2_FC value greater than 1; (3) within the top ten DEGs when lists of genes were ordered by log_2_FC; (4) non-secreted proteins to be identifiable by subsequent immunofluorescence experiments; (5) specificity of the transcript for vascular ECs by visual inspection of feature plots; and (6) consistent expression across the same specialised EC subtype across three or more of the datasets used to generate the scRNA-seq atlas, examined using a dot plot. *SPP1* level was additionally analysed between other non-ECs from control and ADPKD kidneys in a cell type-specific manner using the FindAllMarkers function, as applied to the original annotations from the complete (Muto et al., 2022) dataset. The EC_PKD_ module score was calculated using the AddModuleScore function to the top 50 DEGs, ordered by log_2_FC value, within the EC_PKD_ cyst cluster that were shared between the scRNA-seq and snRNA-seq datasets.

#### Trajectory analysis with pseudotime

To infer lineage trajectories and perform pseudotime analysis, we used the monocle3 package in R (Cao et al., 2019). A cell_data_set object was created from the EC atlas derived from the snRNA-seq dataset of ADPKD human kidneys (Muto et al., 2022). The UMAP embeddings and cluster annotations calculated in the Seurat object as above were transferred as metadata to the cell_data_set object. The *k-*value for nearest neighbours was set at 10 and partition_qval was set as 0.05. The order_cells function was used to select root nodes along the inferred lineage trajectory line to calculate pseudotime.

#### Assessing microvascular subsets in a mouse model of PKD

To compare our findings in human to a mouse model of PKD, we utilised previously published snRNA-seq data of a genetically induced mouse model of PKD. In this mouse model, *Pkd1* deletion occurs in *Pax8^+^* nephron lineages in a doxycycline-dependent manner (Muto et al., 2024). The dataset was downloaded from the GEO database (GSE268494), and the original annotations was used to subset the endothelial cluster. Thereafter, we performed subclustering analysis as above in an iterative fashion to remove non-ECs. Subcluster identity was assigned based on orthologous marker genes identified for human data as above and comparisons with published mouse scRNA-seq analyses of kidney endothelium (Barry et al., 2019; Dumas et al., 2020). *Spp1* expression was analysed across blood endothelial subtypes using a violin plot.

### Mouse *in vivo* experiments and imaging

#### Husbandry

Animal experiments were performed according to the Animal Research: Reporting of *In Vivo* Experiments (ARRIVE) guidelines for reporting animals in research (Percie du Sert et al., 2020). To mimic the slow temporal progression of human ADPKD (Sieben and Harris, 2023), we utilised mice carrying a p.R3277C (RC) hypomorphic allele of *Pkd1* (Hopp et al., 2012), maintained on a C57BL/6J background for at least five generations. Mice carrying the RC allele in heterozygosity (*Pkd1^+/RC^*) were mated, and the progeny were genotyped as previously described (Hopp et al., 2012) to generate wild-type (*Pkd1^+/+^*) or homozygous (*Pkd1^RC/RC^*) mice. To generate *Pkd1^RC/RC^* embryos and littermate controls at E18.5, heterozygous mice were time-mated in the evening, designating the following morning as E0.5 if a copulation plug was detected. Mice were maintained up to 12 months of age and were sacrificed using terminal anaesthesia and death confirmed using cervical dislocation. Throughout the study, all adult mice used in the study were male, to exclude the possibility of sex-based differences in vascularisation, renal perfusion or progression of cystic disease (Arroyo et al., 2021).

#### Surrogate markers of renal function

Kidney-to-body weight ratio and BUN were utilised as surrogate markers for murine renal function and analysed at multiple timepoints up to 12 months of age. Body weight was measured prior to sacrifice, and weight of both kidneys was determined after sacrifice and harvesting of organs by midline laparotomy. BUN assays were performed using a commercially available kit (BioAssay Systems) as per the manufacturer's instructions.

#### MRI configuration

MRI protocols were applied to quantify kidney structure and RBF. Prior to MRI, mice underwent anaesthetic induction using 4% isoflurane in 0.8 l/min medical air and 0.2 l/min O_2_. Following induction and weighing, mice were constrained into the MRI cradle in a supine position using electric tape. Anaesthesia was maintained during the acquisition by reducing isoflurane concentration to 2% in 0.4 l/min medical air and 0.1 l/min O_2_, with a scavenger pump inside the magnet bore to prevent isoflurane build-up. Body temperature was maintained at 37±0.5°C using heated water tubing and monitoring using a rectal probe, and breathing rate was maintained between 90-100 repetitions/min and measured using a respiration pad (SA Instruments). Images were acquired using a 9.4 T Bruker MRI system (BioSpec, 94/20 USR) with a horizontal bore and a 440 mT/m gradient set (BioSpec, B-GA 12S2; outer/inner diameter, 205 mm/116 mm). RBF signal transmission was performed using an 86 mm volume coil, and reception was carried out with a 2×2 four-channel ^1^H receive-only mouse cardiac surface coil (RAPID Biomedical GmbH).

#### Anatomical imaging using T_2_-weighted MRI

To perform *in vivo* anatomical imaging of the kidneys of *Pkd1^+/+^* or *Pkd1^RC/RC^* mice, respiratory-triggered, fat-saturated T_2_-weighted sequences were acquired with a T_2_ TurboRARE sequence (fast-spin echo, Paravision v6.0.1; field of view, 20 mm×30 mm; matrix size, 200×300; RARE factor 4; averages, 4; repetitions, 1; effective echo time, 15 ms; repetition time, 1500 ms; slice thickness, 0.5 mm). This enabled us to differentiate cortex from medulla and facilitated positioning of subsequent RBF measurements.

#### Imaging of RBF using ASL

RBF was quantitatively mapped using a fat-saturated flow alternating inversion recovery (FAIR) pulsed ASL with an echo planar imaging (EPI) readout (field of view, 20 mm×30 mm; matrix size, 100×150; repetitions, 10; effective echo time, 16,000 ms; repetition time, 20.15 ms; dummy scans, 2; imaging slice thickness, 2 mm; segments, 2). For quantification of RBF, an optimised inversion time of 2000 ms was used (Hueper et al., 2014). Slice-selective inversion pulses were applied in the coronal plane matching the imaging slice with a bandwidth of 5200 Hz and thickness of 8 mm. Voxel-wise T_1_ and M_0_ values were also acquired by obtaining EPI data at multiple inversion time (TI) values. For this acquisition, only non-selective inversion was applied using identical parameters as for ASL. Multiple TI values were arrayed within a single sequence: TI=[30, 50, 100, 150, 200, 300, 500, 1000, 1500, 2500, 5000, 8000 ms].

#### Analysis of MRI data

Regions of interest (ROIs) were drawn manually on control (M_C_, non-selective) images to cover the renal cortex or medulla of each kidney, guided by T_2_-weighted imaging. T_1_ and M_0_ values for each voxel within the ROIs were determined using the T_1_ map EPI images. The averaged, multi-TI, non-selective data (M_C_) were fit to a simple inversion recovery model: M_C_=M_0_ (1–2^−TI/T1^). Control (non-selective) and labelled (slice-selective) ASL images were averaged across the ten repetitions. For each ASL image pair, for each voxel within the ROI, the mean M_C_ was subtracted from the mean slice-selective value to provide the perfusion-weighted signal, ΔM. Voxel-wise values of renal perfusion within the ROI were then quantified from the ASL data using a previously described kinetic model (Kim, 1995; Kwong et al., 1995), which incorporates the averaged T_1_, M_0_ and ΔM values. The mean average RBF value from each mouse was reported, across cortex and medulla. T_2_-weighted imaging was used to guide manual selection of ROIs within the cortex to assess RBF within regions devoid of macroscopically overt cysts.

### 3D imaging and analysis of the kidney and its blood microvasculature

#### Harvesting and storage of kidney tissues

For validation of scRNA-seq data, healthy human adult kidney tissue was obtained from three deceased organ donors who were not suitable for transplantation retrieved by a UK National Organ Retrieval Services team. Demographics and clinical metadata for these individuals have been described previously (Jafree et al., 2022 preprint). For validation of snRNA-seq data, tissue was acquired from two individuals with ADPKD derived by the transplant surgical team. Once harvested, the tissues were pseudo-anonymised and incubated overnight in Belzer University of Washington Cold Storage Solution (Bridge to Life Europe, London, UK) at 4°C, before random subsampling into ∼2 mm^3^ pieces. Intact mouse kidney at 3 months of age in *Pkd1^+/+^* or *Pkd1^RC/RC^* mice was collected after MRI and kidney weight assessed after sacrifice. All kidney tissues were incubated in 4% (w/v) paraformaldehyde (Sigma-Aldrich), made up in 1× phosphate buffered saline (PBS), at 4°C overnight. All tissues were then washed and stored in 1× PBS with 0.02% (w/v) sodium azide.

#### Immunofluorescence of healthy human kidney sections for marker validation

Human kidney donor tissues were dehydrated in ethanol, embedded in paraffin and cut into 5 μm sections utilising a rotating blade microtome. Antigen retrieval was performed using Tris-EDTA buffer and endogenous peroxidase quenched using 1.6% hydrogen peroxide. Slides were blocked using CAS-Block (Thermo Fisher Scientific) for 30 min before incubation overnight with the following primary antibodies: mouse monoclonal anti-CD31 (Aligent, GA610, clone JC70A; 1:50), rabbit polyclonal anti-SSUH2 (Atlas Antibodies, HPA049777; 1:100), rabbit polyclonal anti-FBLN2 (Thermo Fisher Scientific, PA5-51665; 1:200) and rabbit polyclonal anti-GPM6A (Proteintech, 15044-1-AP; 1:100). Alexa Fluor-conjugated secondary antibodies (Thermo Fisher Scientific) were used for indirect immunolabelling at a concentration of 1:200, including donkey-anti mouse 546 and donkey anti-rabbit 546. Stained slides were mounted for imaging by Kohler illumination on a Zeiss Axioplan 2 scope.

#### Wholemount immunofluorescence and optical clearing

Human ADPKD kidney tissue samples were randomly selected from 2 mm^3^ pieces, with two samples used per patient. *Pkd1^+/+^* or *Pkd1^RC/RC^* mouse kidneys were manually cut into 200 μm (E18.5) or 500 μm (3 months) slices in axial cross-section. The tissues were then subjected to a wholemount immunostaining protocol (Jafree et al., 2020) formulated by adaptations to iDISCO (Renier et al., 2014) and vDISCO (Cai et al., 2019) protocols. Unless otherwise stated, the following steps were performed at room temperature, all agitated on a flat shaker with incubation volumes of 1 ml, and reagents were purchased from Sigma-Aldrich. Kidney tissues were dehydrated in increasing concentrations of methanol (50, 70%) in double distilled (dd)H_2_O for 30 min per step, before bleaching in absolute methanol with 5% (v/v) of 30% hydrogen peroxide solution overnight at 4°C. The next day, tissues were rehydrated in the methanol series, followed by incubation in 1× PBS for 30 min. Kidneys were permeabilised and blocked with antibody solution (0.09 g trans-1-acetyl-4-hydroxy-L-proline, 0.033 g methyl-β-cyclodextrin, 0.02450 g sodium azide and 245 μl Triton X-100 per 50 ml of 1× PBS) with 6% donkey serum and 10% dimethyl sulfoxide (DMSO) overnight at 37°C, before incubation in antibody solution with 3% donkey serum and 5% DMSO with primary antibodies for 3 days at 37°C. Primary antibodies and labels used for wholemount staining in humans included mouse monoclonal anti-CD31 (1:50) and rabbit polyclonal anti-SPP1 (Abcam, ab8448; 1:100). Rabbit polyclonal anti-SPP1, rat monoclonal anti-CD31 (BD Biosciences, 557355, clone MEC 13.3; 1:50), rat monoclonal anti-endomucin (EMCN, Santa Cruz Biotechnology, sc-65495, clone V.7C7; 1:50) and Dylight 649-conjugated *Lycopersicon esculentum* (Tomato) lectin (Vector Laboratories, DL-1178-1; 1:100) were used on mouse kidney slices. All tissues were then washed in wash solution (0.2 ml of 10 mg/ml heparin stock, 0.4 ml Tween 20 and 0.01 g sodium azide per 200 ml of 1× PBS) four times for 1 h per step, before incubation in antibody solution with 3% donkey serum and 5% DMSO with Alexa Fluor-conjugated secondary antibodies overnight at 37°C. All tissues were further washed in wash solution four times for 1 h per step before dehydration in 50%, 70% and 100% methanol. Optical clearing was performed as previously described (Jafree et al., 2020) using BABB (a 1:2 solution of benzyl alcohol and benzyl benzoate). Immunostained and dehydrated kidney tissues were equilibrated in a 1:1 solution of methanol:BABB, before incubation in BABB until full optical clearance.

#### Confocal microscopy

To capture high-resolution 3D images of the blood microvasculature, human ADPKD kidney tissue samples or *Pkd1^+/+^* or *Pkd1^RC/RC^* mouse kidney slices were placed between a large coverslip and cover glass, supported by an O-Ring (Polymax, Bordon, UK) made from BABB-resistant rubber, as described previously (Jafree et al., 2020). Confocal images were acquired on an LSM880 upright confocal microscope (Carl Zeiss), with 10×/NA 0.5 W-Plan Apochromat water dipping objective [working distance (WD)=3700 μm]. Using 488, 561 or 633 nm lasers for excitation, the vasculature was imaged in three or more randomly selected non-overlapping ROIs. In mouse samples, cortex and medulla were imaged separately, structurally discriminated by autofluorescent signal using the 488 nm laser. Gallium arsenide phosphide (GaAsP) internal and external detectors were used for high sensitivity. Image *z*-stacks were taken through up to 2 mm of tissue and exported as CZI files.

#### Lightsheet fluorescence microscopy

Images of cyst volume and distribution in 3-month-old *Pkd1^RC/RC^* mouse kidney slices were acquired using an Intelligent Imaging Innovations (3i) Cleared Tissue Light Sheet (CTLS) microscope. BABB-cleared samples were immersed in ethyl trans-cinnamate (ECi; 99%) for imaging. The glass imaging chamber was filled with ECi with the sample glued to a custom 3D-printed spoon using ultraviolet-activated glue (UV Bonding). The spoon was magnetically fixed to the stage, and the sample was immersed in the glass chamber with ECi. Illumination was provided by two 5×/0.14 NA air objectives (Edmund Optics, 5X Mitutoyo Plan Apo Infinity Corrected Long WD Objective, #46-143) either side of the glass imaging chamber, and the laser beam was swept perpendicularly to the detection objective to create a lightsheet from either side. A spatial light modulator was used to adjust for chromatic aberrations, as well as providing as many angles for the light as possible to reduce shadowing. For detection, a 1.0×/0.25 NA air objective (Carl Zeiss, PlanNeoFluar Z 1.0×/0.25 NA) was used, followed by a zoom module (Carl Zeiss, Axio Zoom.V16) to change the lateral pixel dimensions on the sCMOS camera, with a zoom of 6.5×, resulting in a lateral pixel dimension of 1 μm. The lightsheet focus was swept horizontally across the sample over five equally spaced positions, and an image was reconstructed to keep only the best axially focused information, with an axial resolution of 7 μm. A 488 nm laser was used to image tissue structure. A quad-band emission filter was placed before the camera (Semrock, FF01-446/523/600/677-25). Images were taken and exported as .sldy files.

#### Segmentation and analysis of cysts within mouse kidneys

Lightsheet images of 3-month-old *Pkd1^RC/RC^* mouse kidney slices were imported as .sldy files into FIJI (v2.1.0) (Schindelin et al., 2012). A 3D gaussian blur was applied to smooth the image stack before the Li thresholding was used to generate a binarised mask of the kidney. The mask was duplicated, and on one duplicate, the Fill Holes function was used within the MorphoLibJ toolkit (Legland et al., 2016) to generate a filled mask of the kidney parenchyma. The original binarised mask, with cysts included, was subtracted from this image to generate a mask containing cysts only. Thereafter, the binarised cysts and the filled binarised kidney were saved as TIFF stacks and exported to IMARIS (Bitplane, v8.2). Within IMARIS, the Isosurface Rendering tool was used to semi-automatically segment the cysts and kidney volume as previously described (Jafree et al., 2019), before manual inspection and removal of rendered structures corresponding to the renal pelvis and arterial vasculature. Total cyst number, cyst volume and kidney volume were then derived from the Statistics toolkit.

#### Segmentation of kidney blood vasculature

Confocal microscopy stacks were imported as CZI images into FIJI. The rolling-ball algorithm was used to subtract background from the images before the image was re-scaled as isometric. For confocal *z-*stacks of 3-month-old *Pkd1^RC/RC^* mouse kidney slices, the greyscaleClosingSphere function was then applied within the CLIJ2 toolkit (Haase et al., 2020), before the Tubeness plugin was used to enhance filamentous structures of the vasculature. Thereafter, the 3D Simple Segmentation plugin (Ollion et al., 2013) was leveraged to generate binarised masks of the vasculature in 3D. Conversely, after background subtraction and re-scaling of E18.5 kidney slices, three randomly selected non-overlapping regions of interest within the cortex and three within the medulla of each kidney were cropped. A 3D gaussian blur was applied (sigma=2 in all dimensions) to each image subregion before auto-thresholding using the ‘mean’ configuration. Thresholding was manually fine-tuned so that all the vasculature was visibly captured, resulting in a binarised mask. The mask was then further processed using the ‘remove outliers’ function to remove noise within the object with a pixel size of less than 3. The ‘close’ function was applied, and the binary mask was dilated and eroded once to ensure continuity of the vessel segments within the binarised mask.

#### Geometric analysis of kidney blood vasculature

The images resulting from our segmentation pipeline were used for analysis of vascular branching metrics, or global measures of vascular patterning, as described below. To derive vascular branching metrics from binarised masks, we utilised the open-source VesselVio (v1.1) application (Bumgarner and Nelson, 2022). Analysis was performed as per instructions (https://github.com/JacobBumgarner/VesselVio) in order to quantify geometric properties of the vascular network, including the length and radius of each vessel branch. We also derived the total number of segments within the vascular network and divided this by the total volume of the kidney volume imaged. Here, kidney tissue volume was quantified by generating a binarised mask of tissue autofluorescence using the method described above, before using the 3D Object Counter plugin in FIJI for quantification. This binarised mask of the tissue boundaries was used for fractal and topological measures, as below. Multiple representative ROIs were analysed per kidney, imaging cortex and medulla separately.

#### 3D fractal and topological analysis of the kidney blood vasculature

From binarised image stacks, we also derived global measures of patterning of the vasculature using fractal analysis and topological data analysis approaches. Briefly, fractal and connectivity dimensions were calculated as described previously (Agterberg, 2013). These parameters give measures of the complexity or ‘roughness’ of an object or pattern, with higher values denoting a more complex and connected vasculature structure. Lacunarity is a fractal-based measure of the distribution of gaps within the vasculature network (de Melo and Conci, 2013), with higher values suggesting heterogeneity due to the presence of voids or spatial clustering. For topological data analysis approaches, 3D skeletonised vasculature networks were first extracted from binary vascular image stacks using ImageJ FIJI Skeletonize3D plugin. Next, the persistence diagram was calculated from branch starting and end points using the python gudhi package (version 3.9.0), from which parameters of interest were calculated as previously described (Chazal and Michel, 2021): the first Betti number, representing the number of vascular loops in the dataset; persistence entropy, a measure of the diversity or variability of the persistence values, where a higher value suggests the presence of a wider variety of topological features across different scales; and the average lifetime of topological features across the dataset. Values were calculated independently for each ROI.

#### Quantification of vascular osteopontin expression and distance from cysts

We used FIJI to assess the fluorescence intensity of SPP1 expression in the cortical vasculature of 3-month-old *Pkd1^RC/RC^* mouse kidney slices and wild-type control kidney slices, which had been wholemount immunolabelled for EMCN and SPP1 and cleared in BABB before 3D confocal microscopy. A maximum-intensity projection was generated from each ROI within the kidney cortex. We used the Measure tool to sample across the whole kidney tissue on average and, thereafter, randomly sampled a minimum of ten CD31^+^ vascular branches per ROI. We derived the mean SPP1 fluorescence intensity such that 30-50 individual vessels per mouse were analysed. To examine the spatial relationship between SPP1^+^ vasculature and cysts, individual *z*-sections from each image were inspected, and the Measure tool was used to quantify the distance between 40 SPP1^+^ CD31^+^ vessels and the nearest cyst boundary, demarcated by SPP1. Distances were binned at 50 μm increments for the cyst boundary, and the number of SPP1^+^ CD31^+^ vessels per bin was quantified across seven ROIs pooled across *n=*3 *Pkd1^RC/RC^* mouse kidneys at 3 months.

#### Sample size and statistics

Based on prior experiments using mouse models of PKD (Hopp et al., 2012; Huang et al., 2016), five to eight mice have been sufficient to assess differences between BUN and kidney-to-body weight ratio. Owing to its sensitivity (Jafree et al., 2019), and based on empirical assessment from a small cohort of three mice per group, we required only four mice per group for sufficient statistical power of 3D vascular analysis. For ASL measurements, we performed pilot experiments of three mice per group to determine that four to six mice would be required to demonstrate the differences observed in RBF. Apart from single-cell transcriptomic analysis described above, all statistics were performed in Prism (v9.5, GraphPad Software). Continuous data were assessed for normality using Shapiro–Wilk tests, and Brown–Forsythe tests were used to assess equality of variance. We used unpaired two-tailed Student's *t*-tests to compare *Pkd1^RC/RC^* and *Pkd1^+/+^* mice for each parameter, with one-way ANOVA and Tukey's pairwise multiple comparisons where more than one timepoint, or region of kidney, was compared. Statistically significant differences were deemed to be *P*≤0.05 and are presented as mean differences, s.e.m. and 95% c.i. for each difference.

## Supplementary Material

10.1242/dmm.052024_sup1 Supplementary information

Table S1. List of differentially expressed genes within the scRNA-seq atlas of kidney endothelial cells. Differential expression analysis was performed using the Wilcoxon rank sum test. Tables are presented with gene names, *p* value (p_val), log_2_FC (avg_log2FC), percentage of cells within the cluster expressing the gene of interest (pct.1), percentage of cells across all other clusters expressing the gene of interest (pct.2), adjusted *p* value (p_val_adj) and name of cell type (cluster).

Table S2. List of differentially expressed genes within the snRNA-seq atlas of ADPKD kidney endothelial cells. Differential expression analysis was performed using the Wilcoxon rank sum test. Tables are presented with gene names, *p* value (p_val), log_2_FC (avg_log2FC), percentage of cells within the cluster expressing the gene of interest (pct.1), percentage of cells across all other clusters expressing the gene of interest (pct.2), adjusted *p* value (p_val_adj) and name of cell type (cluster).

Table S3. List of genes differentially expressed by EC_PKD_ within the snRNA-seq atlas of ADPKD kidney endothelial cells. Differential expression analysis was performed using the Wilcoxon rank sum test. Tables are presented with gene names, *p* value (p_val), log_2_FC (avg_log2FC), percentage of cells within the cluster expressing the gene of interest (pct.1), percentage of cells across all other clusters expressing the gene of interest (pct.2), adjusted *p* value (p_val_adj) and name of cell type (cluster). The first tab in the Excel file contains genes which are expressed, on average, at higher levels compared to other EC types. The second tab contains genes which are expressed, on average, at lower levels compared to other EC types.

## Data and resource availability

Scripts for analysis of single-cell genomics data have been made publicly available at the following GitHub repository: https://github.com/daniyal-jafree1995. Raw and processed count matrices for the scRNA-seq and snRNA-seq studies are available from the original studies from which they were derived. Imaging data, including confocal microscopy and MRI outputs, are available from the corresponding author upon reasonable request.
