# Supplementary material for: Microvascular aberrations found in human polycystic kidneys are an early feature in a Pkd1 mutant mouse model
Source: Dis Model Mech. 2025 Apr 28;18(4):dmm052024. doi: 10.1242/dmm.052024 (PMC12067086; doi:10.1242/dmm.052024)
Supplement: Supplementary information [file dmm-18-052024-s1.pdf]

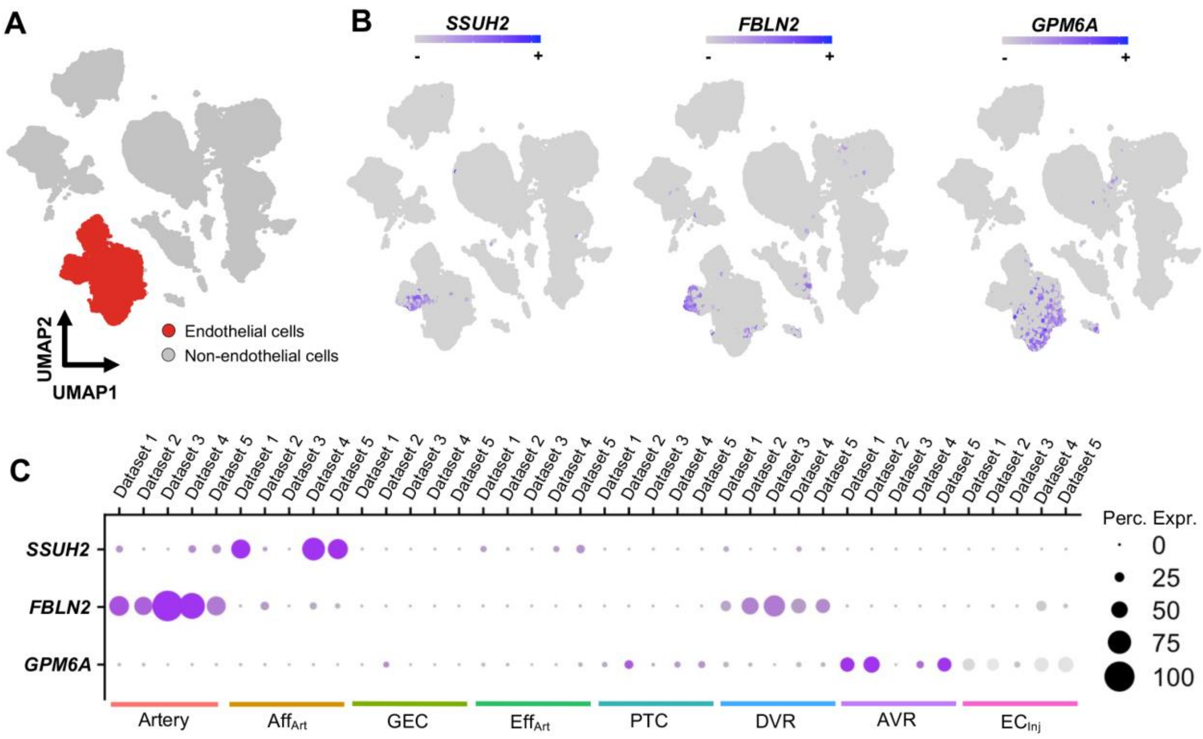

Fig. S1. Profiling marker gene expression of vascular endothelial cell subsets across the human kidney blood endothelial single-cell atlas.

(**A**) Uniform manifold approximation and projection (UMAP) of a kidney single-cell RNA sequencing (scRNA-seq) atlas, with the *CDH5*$^{+}$ *FLT1*$^{+}$ endothelial cell (EC) cluster shown in red. (**B**) Feature plots showing expression of *SSUH2*, *FBLN2* and *GPM6A* across the dataset, demonstrating restricted expression within the endothelial cluster. (**C**) Dot plot showing consistent expression of above selected marker genes across the five individual datasets used to construct the atlas.

Disease Models & Mechanisms • Supplementary information

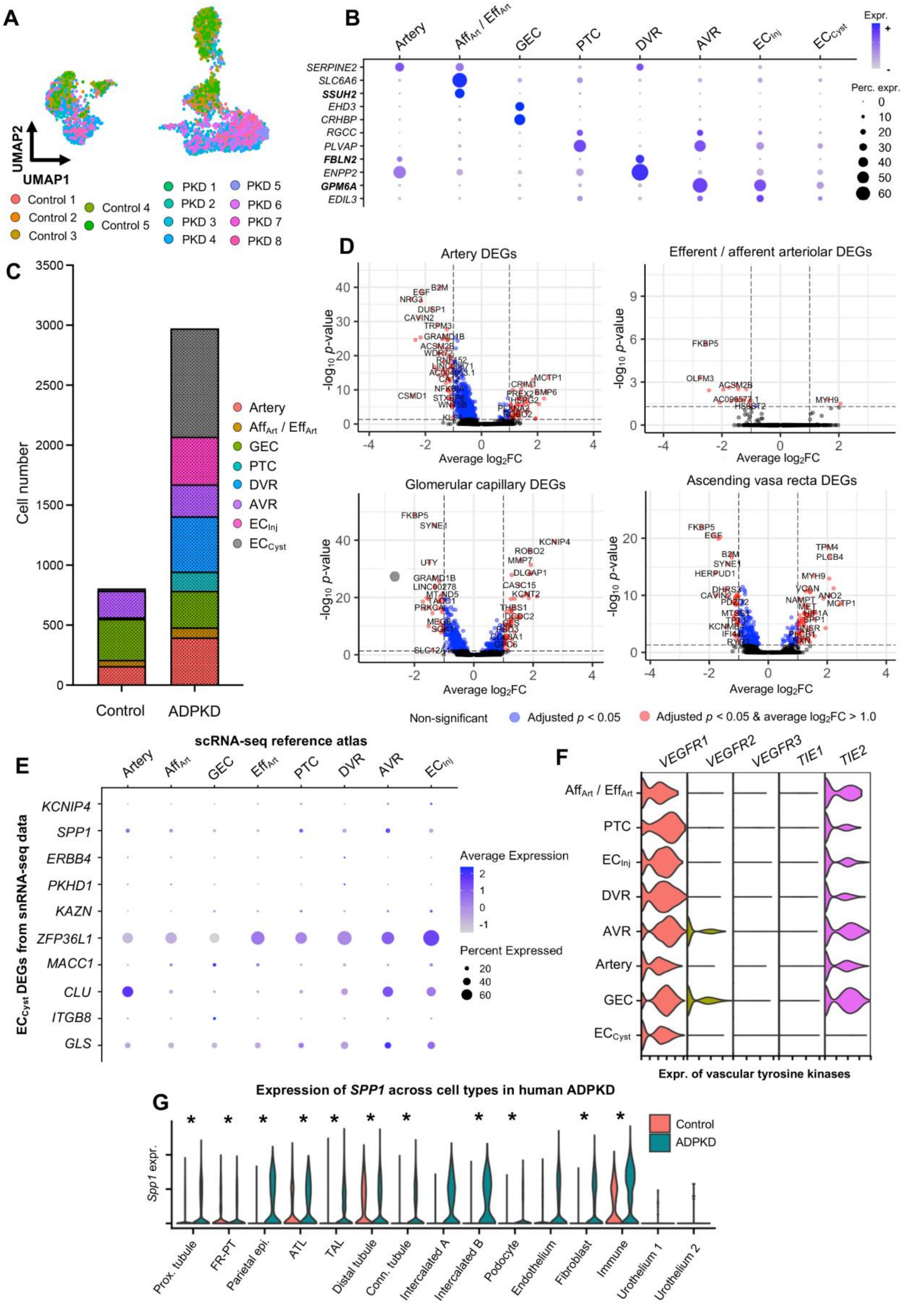

Disease Models & Mechanisms • Supplementary information

Fig. S2. Annotation and characterization of blood endothelial cells in human ADPKD kidneys.

(**A**) Individual samples used to construct the endothelial cell (EC) snRNA-seq dataset in ADPKD, including 5 control kidneys and 8 kidneys with ADPKD. (**B**) Dot plots showing top 2-3 marker genes per cluster, with selected candidates *SSUH2*, *FBLN2* and *GPM6A* annotating the corresponding EC types within this new dataset. (**C**) Bar chart showing relative abundance of EC types across conditions. Injured ECs (EC_{Inj}) and a novel discrete population, termed EC_{PKD}, are enriched in ADPKD samples. (**D**) Violin plot showing differential expression analysis of different EC types between conditions. Each point represents a gene, with red points being differentially expressed and statistically significant, blue not meeting criteria for significance and black not differentially expressed. **(E)** Dot plot of DEGs identified in EC_{PKD}, analyzing their EC-specific expression within the prior scRNA-seq kidney endothelial cell reference atlas. **(F)** Violin plot of vascular tyrosine kinases expressed by EC_{PKD}. These include members of the vascular endothelial growth factor receptor (VEGFR) family and members of the tyrosine kinase with immunoglobulin like and EGF like domains (TIE) family. (**G**) Examination of *SPP1* expression in non-EC types. Data are visualized as a violin plot. The asterisks denote a significant difference ($p < 0.05$) in individual Wilcoxon rank sum tests.

Disease Models & Mechanisms • Supplementary information

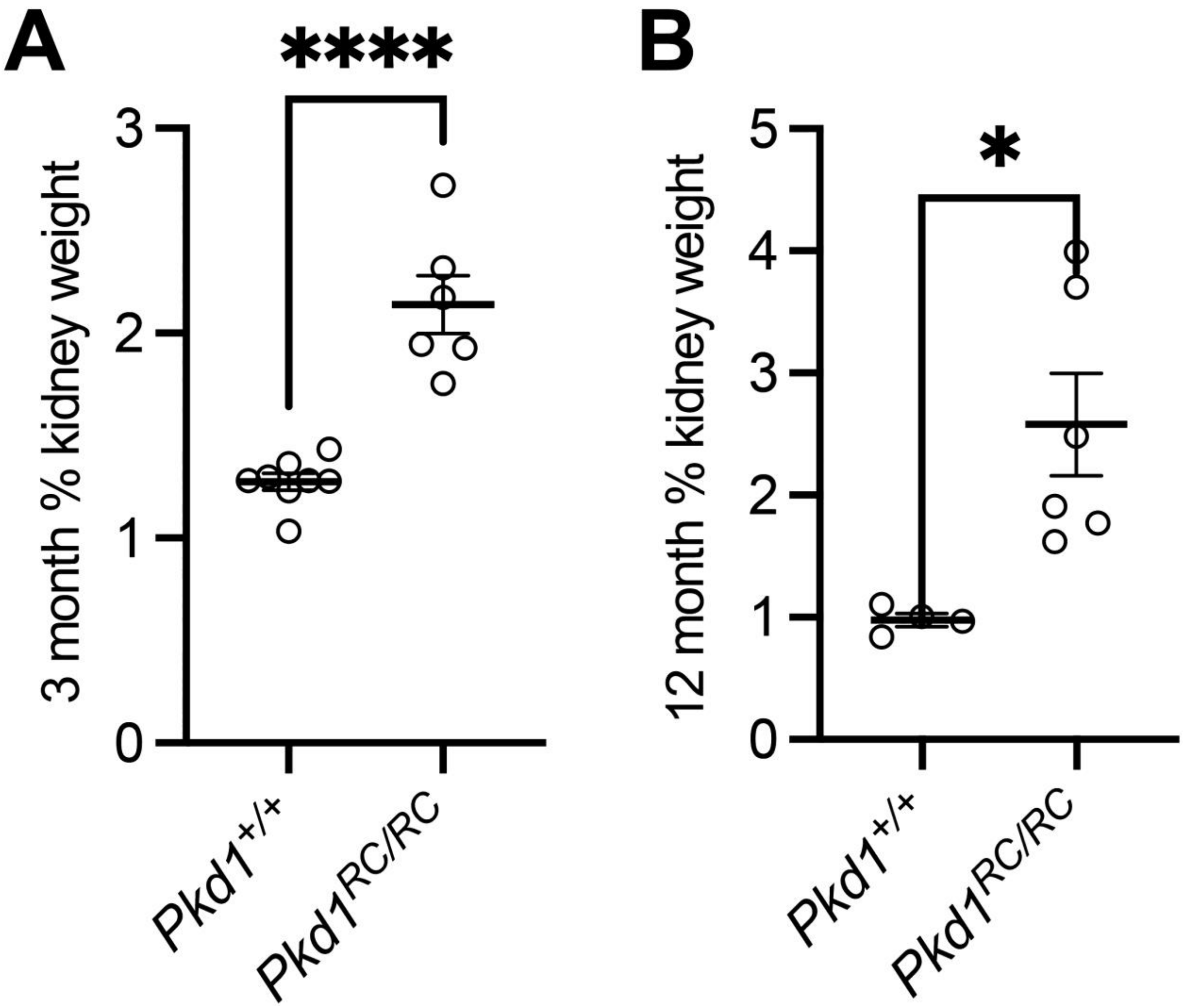

Fig. S3. Characterisation of kidney to bodyweight ratios in a mouse model of ADPKD.

(A-B) Assessment of kidney to bodyweight ratio at 3 months (**A**) and 12 months (**B**) in *Pkd1*$^{RC/RC}$ as compared to *Pkd1*$^{+/+}$ mouse kidneys. Student's *t* test demonstrated a statistically significant increase in mutant as compared to wildtype mice at 3 months (mean difference in 0.87 ± 0.13% kidney weight, 95% CI = 0.58-1.15, p < 0.0001) and at 12 months (mean difference in 1.6 ± 0.53% kidney weight, 95% CI = 0.39-2.81, p = 0.016). Each point on the graph represents an individual mouse analysed. *: p < 0.0332, **: p < 0.0021, ***: p < 0.0002, ****: p < 0.0001.

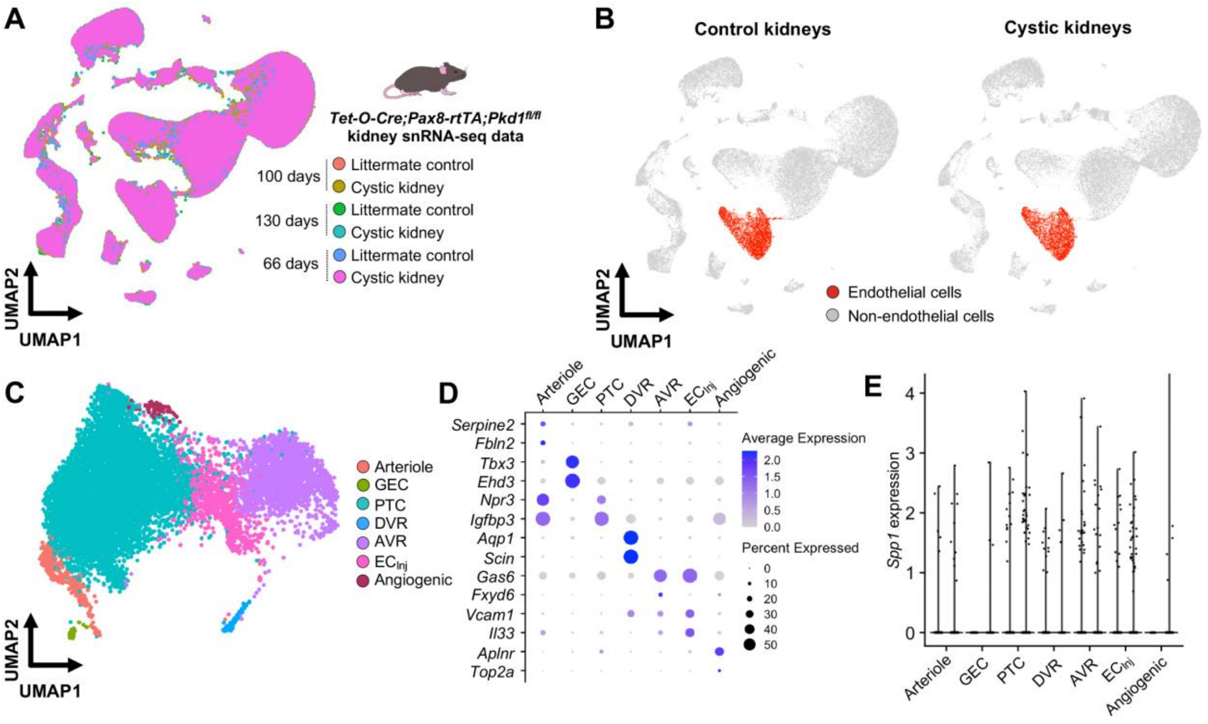

Fig. S4. Transcriptomic analysis of the endothelium in mice with inducible *Pkd1* deletion within the *Pax8*$^{+}$ nephron lineage.

(**A**) UMAP showing integrated snRNA-seq data of six kidneys (each from a separate mouse) at different timepoints within the *Tet-O-Cre;Pax8-rtTA;Pkd1*$^{fl/fl}$ model of PKD, at 66 (early stage), 100 (intermediate stage) and 130 days (late stage). (**B**) UMAPs partitioned by cystic kidneys or littermate controls showing the EC cluster within the snRNA-seq dataset. (**C**) ECs were subset from the original aggregated dataset and subclustering was performed to identity distinct subpopulations. An EC_{PKD} population was not detected in this data. (**D**) Dotplot showing discrimination of markers of each subpopulation of the microvasculature, with markers derived from our analysis of human data and also from previously published studies. (**E**) Violin plot showing scant expression of *Spp1* within the EC subclusters. Each EC subtype within the plot has paired data with the left corresponding to control kidneys and the right corresponding to mutant kidneys.

Disease Models & Mechanisms • Supplementary information

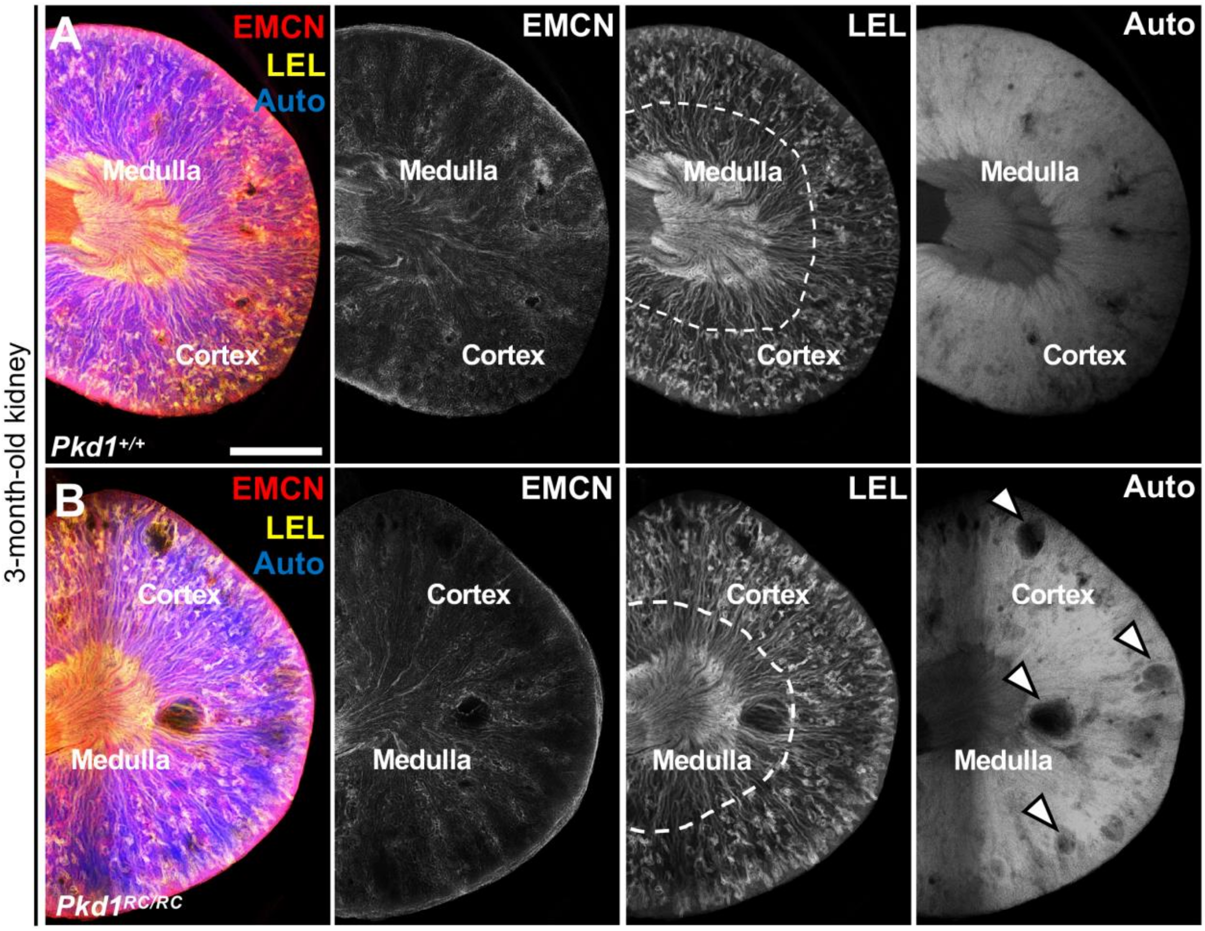

Fig. S5. Characterisation of microvasculature and cysts in kidney cortex and medulla of an ADPKD mouse model at an intermediate disease stage.

(**A-B**) Maximum intensity projection of *Pkd1*$^{+/+}$ (**A**) or *Pkd1*$^{RC/RC}$ (**B**) kidneys stained for endomucin (EMCN) and tomato lectin (LEL). Autofluorescence (Auto) was imaged to capture tissue architecture. The dotted line separated cortex from medulla, clearly demonstrated by the pattern of LEL staining. Arrowheads show cysts within the autofluorescence channel. All images are representative of *n* = 4 mouse kidneys per group. Scale bar = 1 mm.

Disease Models & Mechanisms • Supplementary information

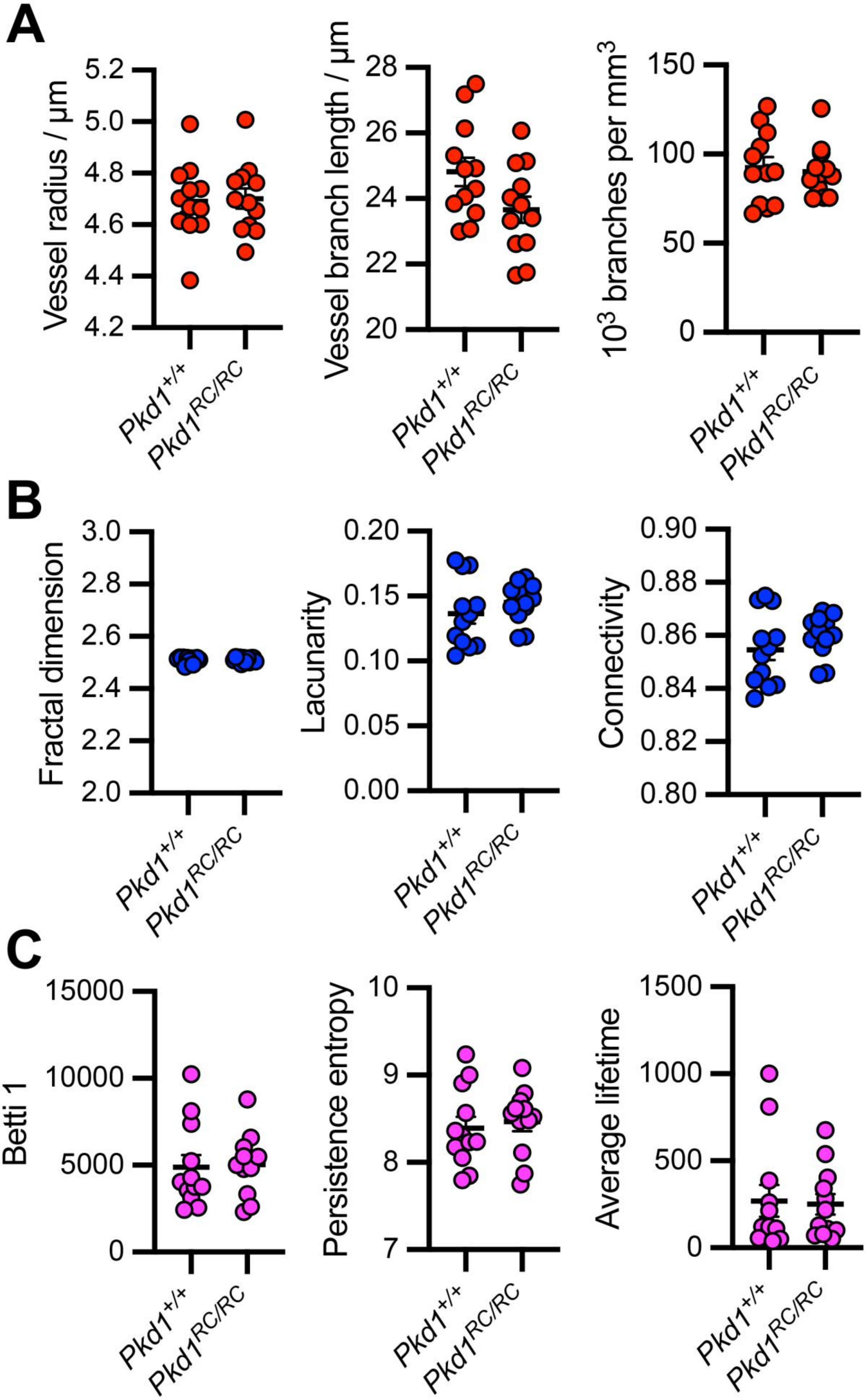

Disease Models & Mechanisms • Supplementary information

Fig. S6. Three-dimensional analysis of the medullary kidney microvasculature in a mouse model of ADPKD at an intermediate stage.

3D geometric analysis (**A**), including vessel radius, length and density (defined as 10^3 vessel branches per mm^3 of tissue), fractal analysis (**B**), including fractal dimension, lacunarity and connectivity and topological analysis (**C**), including Betti 1, persistence entropy and average lifetime of the medullary microvasculature between *Pkd1*$^{+/+}$ and *Pkd1*$^{RC/RC}$ kidneys at 3 months of age. Student's *t* tests demonstrated no significant differences between mutant and wildtype medullary microvasculature in any of these measures. Each point represents an individual region of interest imaged within the medulla and pooled across *n* = 4 mice per group.

Disease Models & Mechanisms • Supplementary information

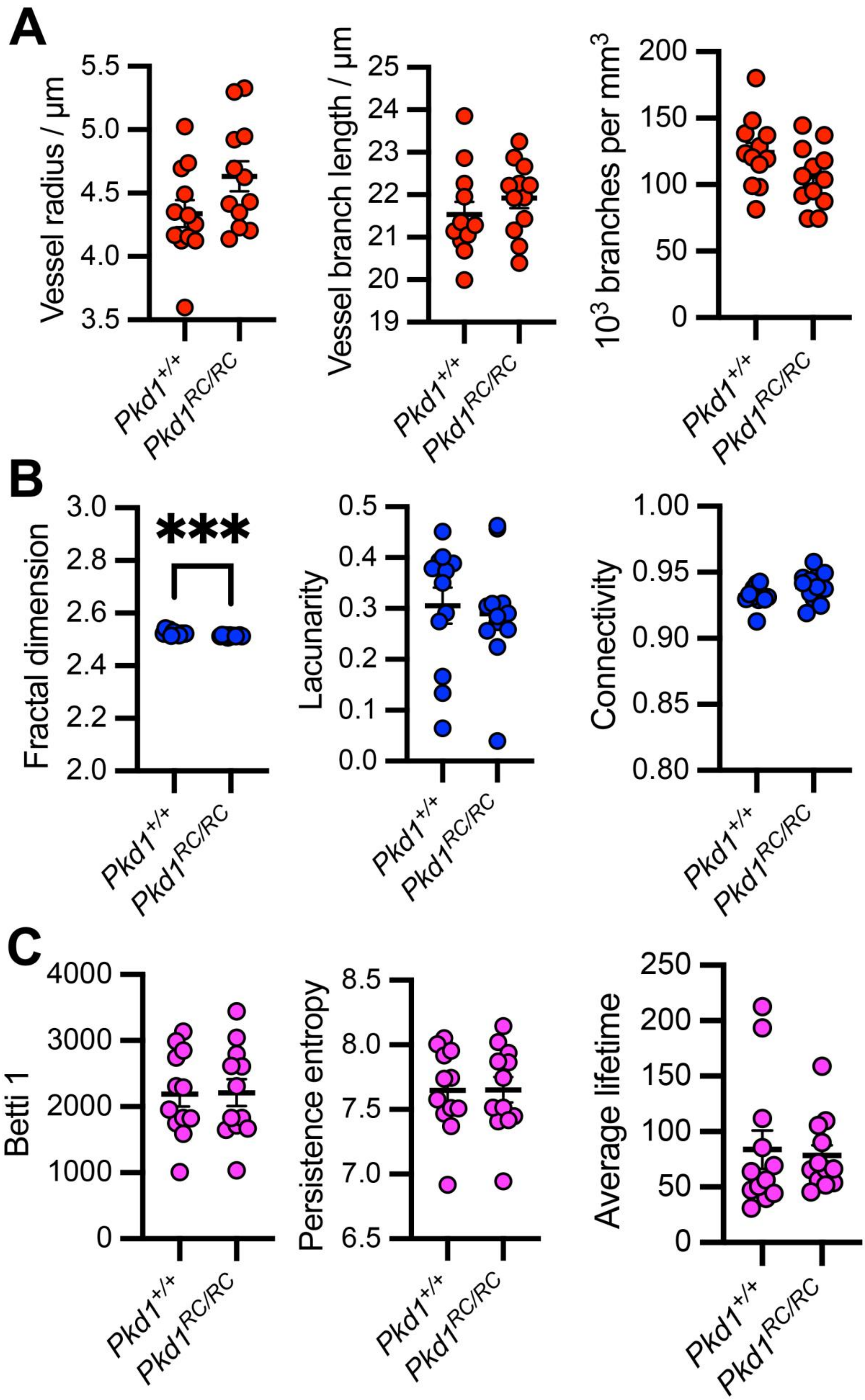
A
Vessel radius / µm
Vessel branch length / µm
10^3 branches per mm^3
B
Fractal dimension
***
Lacunarity
Connectivity
C
Betti 1
Persistence entropy
Average lifetime
Pkd1+/+
Pkd1RC/RC

Fig. S7. Three-dimensional analysis of the medullary kidney microvasculature in a mouse model of ADPKD at a fetal stage.

3D geometric analysis (**A**), including vessel radius, length and density (defined as 10^3 vessel branches per mm^3 of tissue), fractal analysis (**B**), including fractal dimension, lacunarity and connectivity and topological analysis (**C**), including Betti 1, persistence entropy and average lifetime of the medullary microvasculature between *Pkd1*$^{+/+}$ and *Pkd1*$^{RC/RC}$ kidneys at E18.5. Student's *t* tests were performed, and fractal dimension was the only parameter to be significant different between the two groups, which a small decrease in the mutant compared to wildtype medullary microvasculature (mean difference = 0.01 ± 0.0025, 95% CI = 0.005-0.02, p = 0.0004). Each point represents an individual region of interest imaged within the medulla and pooled across *n* = 4 mice per group.

Table S1. List of differentially expressed genes within the scRNA-seq atlas of kidney endothelial cells.

Differential expression analysis was performed using the Wilcoxon rank sum test. Tables are presented with gene names, *p* value (p_val), log_2FC (avg_log2FC), percentage of cells within the cluster expressing the gene of interest (pct.1), percentage of cells across all other clusters expressing the gene of interest (pct.2), adjusted *p* value (p_val_adj) and name of cell type (cluster).

Available for download at
https://journals.biologists.com/dmm/article-lookup/doi/10.1242/dmm.052024#supplementary-data

Table S2. List of differentially expressed genes within the snRNA-seq atlas of ADPKD kidney endothelial cells.

Differential expression analysis was performed using the Wilcoxon rank sum test. Tables are presented with gene names, *p* value (p_val), log_2FC (avg_log2FC), percentage of cells within the cluster expressing the gene of interest (pct.1), percentage of cells across all other clusters expressing the gene of interest (pct.2), adjusted *p* value (p_val_adj) and name of cell type (cluster).

Available for download at
https://journals.biologists.com/dmm/article-lookup/doi/10.1242/dmm.052024#supplementary-data

Table S3. List of genes differentially expressed by EC_{PKD} within the snRNA-seq atlas of ADPKD kidney endothelial cells.

Differential expression analysis was performed using the Wilcoxon rank sum test. Tables are presented with gene names, *p* value (p_val), log_2FC (avg_log2FC), percentage of cells within the cluster expressing the gene of interest (pct.1), percentage of cells across all other clusters expressing the gene of interest (pct.2), adjusted *p* value (p_val_adj) and name of cell type (cluster). The first tab in the Excel file contains genes which are expressed, on average, at higher levels compared to other EC types. The second tab contains genes which are expressed, on average, at lower levels compared to other EC types.

Available for download at
https://journals.biologists.com/dmm/article-lookup/doi/10.1242/dmm.052024#supplementary-data
